# Reducing cognitive demand fails to mitigate choice overload: Multimodal neural and gaze evidence for effort conservation

**DOI:** 10.1016/j.isci.2026.117616

**Published:** 2026-09-24

**Authors:** Seungbeom Seo, Seungji Lee, Nayoung Lee, Sung-Phil Kim

**Affiliations:** 1 Department of Biomedical Engineering, Ulsan National Institute of Science and Technology, Ulsan, South Korea; 2 Department of Electrical Engineering, Ulsan National Institute of Science and Technology, Ulsan, South Korea; 3 Digital Marketing Department, Kyongnam Bank Head Office, Changwon, South Korea; 4 Department of Industrial Engineering, Ulsan National Institute of Science and Technology, Ulsan, South Korea

**Keywords:** choice overload, cognitive effort, effort conservation, fixation-related potentials, eye tracking, decision-making

## Abstract

An overabundance of options can impair decision-making, a phenomenon known as choice overload. Modern platforms use recommendation and filtering tools to mitigate this by lowering cognitive demand, yet whether these strategies actually improve choices remains unclear. We conducted two experiments that manipulated cognitive demand—by presenting high-attractiveness option sets or by providing a shortlist tool—while recording eye-tracking and electroencephalography (EEG) as participants chose from sets of 6–24 options. Reducing demand accelerated decisions but did not improve choice performance as set size increased. Under low-demand conditions, eye-gaze measures revealed narrower visual search and fixation-locked EEG showed shorter event-related potential latencies and weaker occipital alpha suppression, and the three modalities co-varied within individuals, indicating that participants conserved rather than reallocated cognitive effort. These findings demonstrate that simplifying decisions can backfire: when choices feel easier, people disengage rather than optimize, leaving choice overload intact. Effective choice architecture should therefore sustain deliberation, not merely reduce demand.

## Highlights

• Filtering and shortlisting eased choosing but did not reduce choice overload
• Low demand narrowed search: fewer options screened, fewer revisits
• Fixation-locked EEG showed shallower processing of each option under low demand
• Effort is conserved rather than reallocated, therefore choice overload persists

## Graphical abstract

**Figure:**
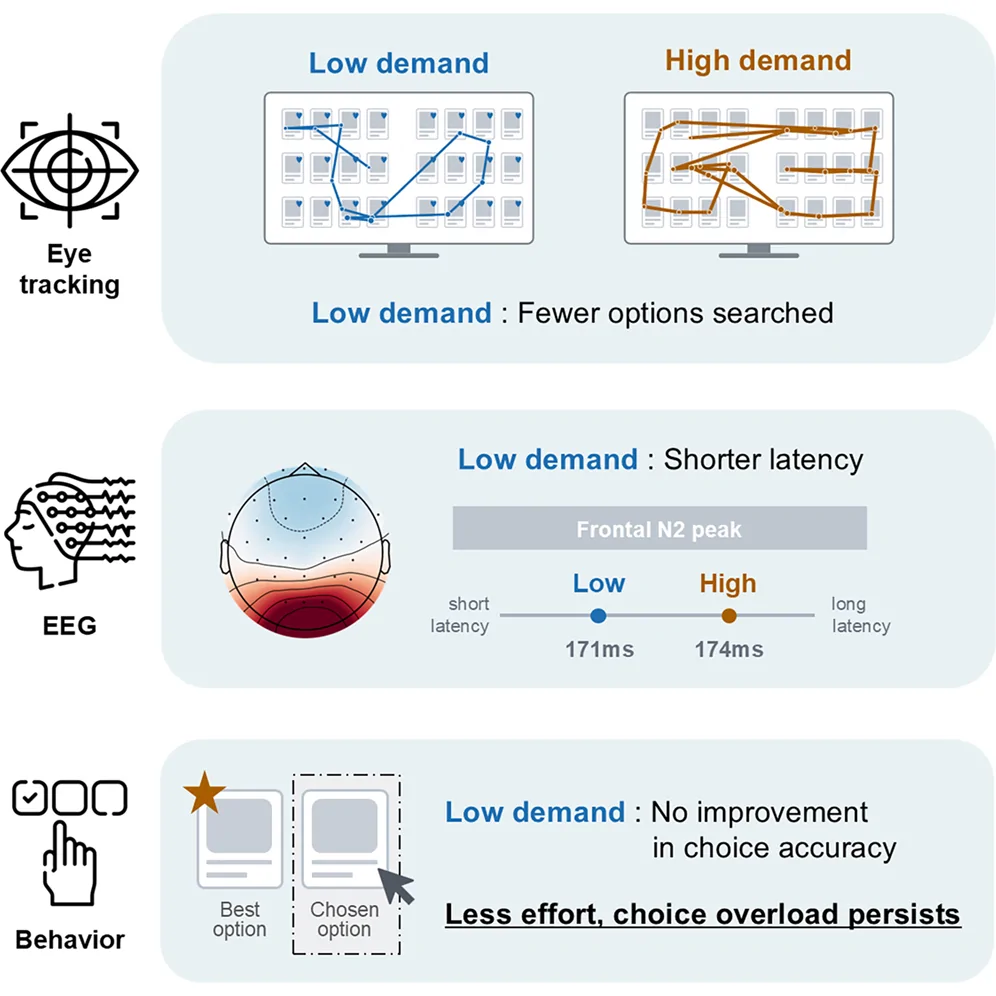

## Teaser

Sensory neuroscience; Cognitive neuroscience

## Introduction

Every day, people face a staggering number of decisions.^1^ With such an abundance of decisions, it may seem that having more options is better. Indeed, larger assortments can increase the sense of freedom and variety, potentially making decision-making more enjoyable.^2^^,^^3^^,^^4^^,^^5^ However, beyond a certain point, an overabundance of options can lead to choice overload, wherein decision-making suffers, producing negative outcomes like stress, regret, or lower satisfaction.^6^^,^^7^^,^^8^^,^^9^^,^^10^^,^^11^ For example, in digital media, an overabundance of content can cause people to spend more time choosing than consuming (e.g., a “Netflix syndrome”), leading to frustration.^12^ In such cases, the cognitive costs of processing many alternatives outweigh the benefits of added variety.^13^^,^^14^^,^^15^^,^^16^^,^^17^^,^^18^ Despite the ubiquity of demand-reduction tools across digital commerce, healthcare, and public policy, the assumption that easing decisions leads to better outcomes has not been rigorously tested with process-level measures.

A useful framework for understanding choice overload is the evaluation-cost model.^13^ As the number of options grows, the mental effort required to evaluate them (cognitive demand) rises sharply, while the marginal benefit of each additional option diminishes. This trade-off suggests that decision-makers would continue examining options only until the expected benefit of doing so no longer justifies the cognitive effort. Neuroscientific evidence supports this notion by showing that reward-related brain regions responded most strongly to moderate assortment sizes, but showed reduced activation for larger choice sets.^19^ Thus, when faced with abundant options, people often downshift their cognitive effort to avoid excessive mental cost.^20^^,^^21^^,^^22^ This tendency to conserve effort can lead to suboptimal decisions, as individuals may not fully explore all options.^23^^,^^24^

Since choice overload stems from an overly taxing evaluation process, a logical remedy is to ease the cognitive burden. Because human attentional and working memory capacity is limited, evaluating a large number of options can quickly overwhelm our cognitive resources.^25^^,^^26^ By reducing the decision-maker’s cognitive demand, people can handle more alternatives without feeling overwhelmed.

In modern consumer environments, two common strategies aim to make choices easier by reducing cognitive demand:

Strategy 1 is preference-based filtering (personalized recommendations): this approach presents the decision-maker with a curated subset of highly attractive options, tailored to individual preferences. By filtering out less desirable choices (e.g., recommending a short list of top-rated movies), the decision becomes easier because all the options are likely to be good, and the stakes of a “wrong” choice are lower. Thus, the cognitive effort needed to compare options is greatly reduced. For instance, Chernev and Hamilton^27^ found that when consumers were offered only uniformly high-quality options, they perceived less risk and were satisfied with the choice more quickly.^27^

Strategy 2 is sequential narrowing of choices: this approach breaks the decision into stages, rather than confronting the chooser with all options at once. A common example is a “wishlist” or bookmarking tool on e-commerce platforms: from a large initial pool, users first mark a subset of favorites, and then make a final selection from that subset. By narrowing the choice set sequentially, the cognitive demand is reduced because each stage involves evaluating fewer options. Importantly, by the final stage, the decision-maker has already evaluated the options during the filtering step, so they are not processing all new information simultaneously. This sequential filtering process allows focus on fewer options at a time, easing cognitive load during the final choice.

However, it remains elusive whether making decisions easier through these industry-driven strategies indeed leads to better choices and prevents overload. On one hand, reducing cognitive demand could enable people to engage more fully with options, avoiding the negative effects of overload. With only high-quality, pre-selected options, individuals might invest their extra mental resources into comparing options more thoroughly, yielding more optimal decisions. On the other hand, simplifying a choice may not guarantee better outcomes. People often act as cognitive misers,^28^ seeking to minimize unnecessary mental work. Therefore, when a task is made easier, decision-makers might respond by exerting even less effort, reasoning that minimal effort is now sufficient. Ironically, strategies designed to alleviate overload could cause people to coast through the decision. If so, any benefit of reduced demand is canceled out by reduced effort investment, leaving the choice overload problem intact or even exacerbated.

Therefore, a key research question is whether making a choice set “easier” prompts individuals to reallocate their spare mental capacity to the task or leads them to conserve effort and drift into a low-effort decision strategy. It is difficult to answer this with traditional self-reports or behavioral measures, which are limited in capturing temporal dynamics in the decision process.^29^ Instead, leveraging neurophysiological indicators of cognitive effort can reveal how people adjust their engagement when a decision is simplified.^30^ Complementing this approach, prior neurophysiological studies of choice overload suggest that excessive alternatives can disrupt early-stage processing and recruit later compensatory engagement.^31^^,^^32^

Huang and Xu^33^ provided initial evidence that high-preference choice sets accelerate decisions without improving performance, and that neural engagement can increase for smaller, harder sets.^33^ However, they did not manipulate cognitive demand through task constraints or decision aids and were limited to two set sizes, precluding systematic examination of how demand reduction interacts with set size across the range relevant to real-world overload. Moreover, without concurrent process measures, the mechanistic pathway linking demand reduction to effort allocation remained unclear.

Here, we address this gap with a multimodal experimental approach that offers three advances. First, we directly manipulate cognitive demand using two ecologically relevant strategies—preference-based filtering and sequential narrowing via a shortlist tool—enabling causal inference about demand-reduction interventions used across industries. Second, we parametrically vary set size (6, 12, 16, and 24 options) to trace how demand reduction interacts with the full range of assortment size. These set sizes were chosen to bracket the range over which choice overload typically emerges, with 6 lying below and 12–24 at or above the thresholds at which overload effects have been reported.^10^^,^^19^^,^^34^ For counterbalancing, each demand condition drew from a fixed 48-item pool—the least common multiple of the four set sizes—so that 6/12/16/24 could be sampled in balanced subsets onto a fixed 3 × 8 grid (16 was used rather than 18 to satisfy this constraint). Third, we combine concurrent eye-tracking and fixation-locked EEG to decompose effort into search-level and item-level neural processing components, providing mechanistic resolution unavailable from behavioral data alone. The logic of this decomposition is that the two modalities index complementary facets of effort. Eye movements reveal where and how broadly attention is deployed across the array—the breadth of search (search-level effort)—whereas EEG epochs time-locked to each fixation index the depth of processing devoted to an individual option at the moment it is foveated (item-level effort). Combining them separates a reduction in how widely one searches from a reduction in how deeply each option is evaluated.

In experiment 1 (E1), we simulated preference-based filtering by manipulating the attractiveness of the choice set, a strategy akin to providing preference-articulated choice set.^35^ Participants chose from sets of options that were either all high-preferred (low-demand condition) or all low-preferred (high-demand condition). This mirrors a personalized recommendation system that boosts option attractiveness to ease decision-making. In experiment 2 (E2), we implemented sequential narrowing. Participants first completed a preliminary bookmarking step to select a handful of favorites from a larger set, and then made a final choice from those bookmarked items. In the low-demand condition, the final choice set was “screened” by their bookmarks, whereas in the high-demand condition, the final set was a control assortment without such pre-selection. Detailed task sequences and demand manipulations are illustrated in Figure 1. We also report manipulation checks confirming the demand conditions (see “Manipulation checks,” later, and Figure S1).

**Figure 1:**
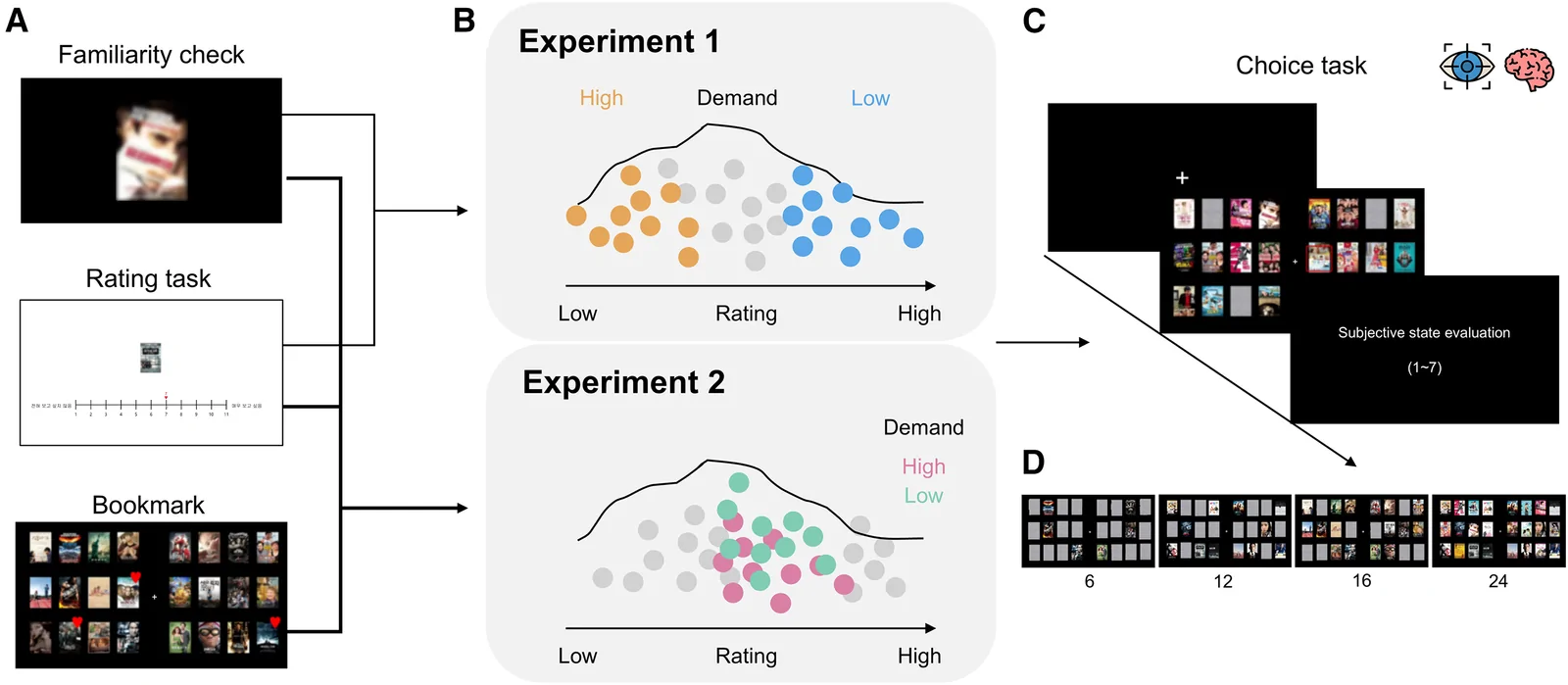
Experimental procedure (related to STAR Methods) (A) Session sequence: a familiarity check, a two-round rating task (11-point scale), an Experiment-2-only bookmarking task, and the choice task with post-choice experience ratings (7-point scale). (B) Demand manipulation. E1 (preference filtering): low-demand sets drew from a high-attractiveness pool (48 top-rated posters), high-demand sets from a low-attractiveness pool (48 bottom-rated posters), with rating variance matched. E2 (sequential narrowing): low-demand (“screened”) sets were anchored on the participant’s bookmarks, high-demand (“control”) sets were drawn from a rating-matched pool with no bookmarks. (C) Single-trial timeline: 2-s fixation, self-paced choice array (mouse click), then four experience questions. (D) Representative choice-array layouts for set sizes 6, 12, 16, and 24 on the fixed 3 × 8 grid. Every set size filled the same fixed 3 × 8 grid, with the unused positions left blank and evenly distributed across the array, so that no set size introduced a spatial bias (verified in Figure S2).

If reducing cognitive demand encourages greater engagement, we expected less decline in decision quality as the set size increases in the low-demand than the high-demand conditions. Conversely, if participants in easier tasks tend to slack off, there would be no improvement in choice outcomes despite the simplification.

To address these questions, we measured not just which options participants chose, but how they made their decisions. We used multi-modal measurements, including eye-tracking to index visual search behavior and electroencephalography (EEG) to gauge neural engagement, on a “moment-to-moment” basis. We derived three eye-gaze measures of visual search (screening ratio, revisit ratio, and dwell bias) and, from EEG epochs locked to each fixation, fixation-related potential (FRP) component latencies and fixation-related occipital alpha suppression as indices of per-item processing effort. Full operational definitions are given in the STAR Methods (quantification and statistical analysis). In brief, longer FRP latencies and stronger alpha desynchronization index more effortful per-item processing. In summary, our use of the demand-reduction strategies and fine-grained effort measures tests whether making choices easier leads to effort reallocation or effort conservation.

## Results

### Manipulation checks

Because our central claim rests on the two interventions genuinely lowering cognitive demand, we asked both whether each manipulation was implemented as designed and whether the resulting effort differences might be explained more simply. The manipulations were realized as intended. In E1 the high-attractiveness (low-demand) pool was rated far higher than the low-attractiveness pool (F[1, 16] = 1,129.6, *p* < 0.001, Figure S1), whereas in E2 the screened and control pools were deliberately rating-matched (mean difference 0.13 on the 11-point scale, Figure S1), so that any demand difference there arose from prior self-evaluation (bookmarking), not option quality. Consistent with lower demand, the low-demand condition also elicited faster decisions (5.85 vs. 7.38 s in E1, t[16] = −5.32, *p* < 0.001, and 5.47 vs. 6.30 s in E2, t[27] = −6.34, *p* < 0.001) and narrower search (screening ratio 0.69 vs. 0.81 in E1, t[16] = −5.50, *p* < 0.001, and 0.69 vs. 0.77 in E2, t[27] = −7.88, *p* < 0.001). Because these measures also index the effort allocation we analyze below, we take them to show that the manipulation shifted processing in the expected direction rather than as independent proof of reduced demand.

One possibility is that within-set rating spread (how far apart the options sit on the rating line), rather than our intended manipulation, drives effort. Two analyses argue against this. First, within-set rating spread did not predict effort. Pooling free-choice trials and removing between-subject differences, the within-subject correlation between a trial’s within-set rating standard deviation and its decision time was near zero (r = −0.07 in E1 and r = −0.003 in E2), as was its correlation with the number of fixations (r = −0.12 in E1 and r = 0.02 in E2), and the same held within each demand condition separately (all |r| ≤ 0.10). Second, the incidental spread difference between conditions points the wrong way for a spread-based account. If the low-demand condition were easier simply because its options were more alike, with a narrower spread, its sets should have shown less spread than the high-demand sets. In E1 they instead showed more (1.07 vs. 0.41, t[16] = 3.29, *p* = 0.005), yet still elicited less effort. Thus the reduction in effort tracks the demand manipulation (option attractiveness in E1, prior evaluation in E2), not the spread of ratings within a set. Critically, as the following sections show, this reduced effort did not translate into better choices, a dissociation that distinguishes effort conservation from the reallocation of freed capacity to more thorough evaluation.

### Experiment 1 (preference-based filtering)

The primary behavioral outcomes were defined at the trial level and summarized within each demand × set size condition. Choice performance quantified the extent to which the selected option approached the best available alternative in the set, based on each participant’s own pre-task ratings; for each trial, we computed the difference between the ratings of the chosen item and the highest-rated item among the displayed options. Decision time was defined as the latency from choice-set onset to the mouse click indicating selection. These trial-wise measures were then averaged across trials within each condition for each participant (see STAR Methods for full operational definitions and preprocessing details).

In E1, choice performance was significantly influenced by both demand condition and set size. Overall, performance was lower in the low-demand than in the high-demand conditions (F[1, 16] = 13.66, *p* = 0.002, η^2^*p* = 0.46). We also observed an overall decline in performance as set size increased (F[3, 48] = 11.07, *p* < 0.001, η^2^*p* = 0.41). Importantly, a significant demand × set size interaction effect (F[3, 48] = 11.89, *p* < 0.001, η^2^*p* = 0.43) indicated that performance in the low-demand condition worsened substantially as set size increased, while performance in the high-demand condition remained largely stable (Figure 2A, left). Thus, making options more appealing did not enable participants to maintain high-quality choices as the assortment grew.

**Figure 2:**
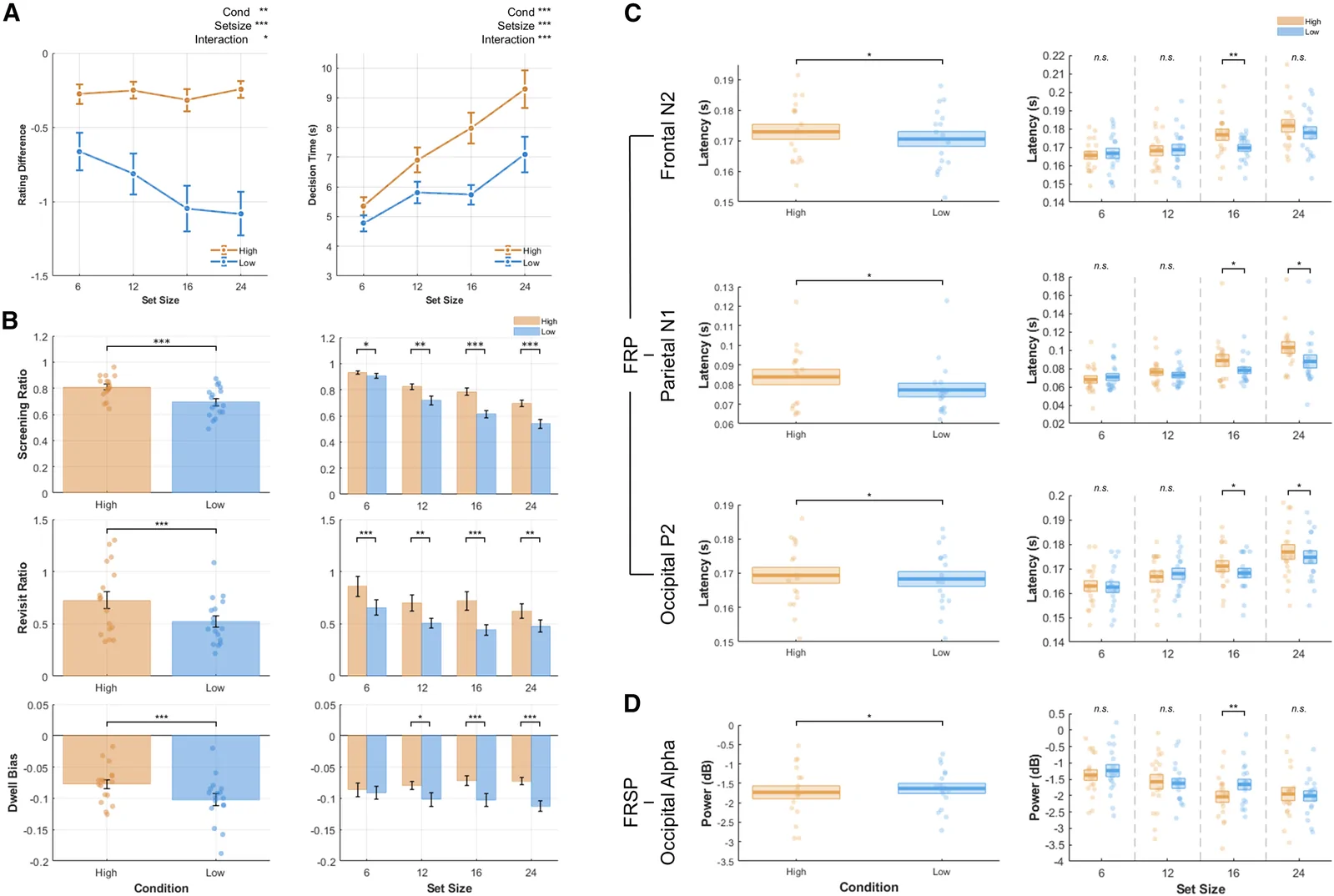
Results of E1 (preference-based filtering) (A) Rating difference between the chosen item and the best-rated item in the set (left) and decision time (right), shown by demand condition (high vs. low) and set size (6, 12, 16, and 24). (B) Eye-tracking measures—screening ratio (fraction of items viewed), revisit ratio (average returns per fixated item), and dwell bias (normalized gaze-time bias)—shown by demand condition (left; individual participants, *n* = 17) and by set size (right). (C) EEG FRP peak latencies. (D) EEG FRSP in the alpha band. In (A and B), error bars denote ±SEM. In (C and D) individual participants are shown (*n* = 17), and boxplots summarize the mean (center line) with boundaries indicating ±SEM. Statistical significance from two-way repeated-measures ANOVA is indicated as: n.s. for non-significant, ∗ for *p* < 0.05, ∗∗ for *p* < 0.01, and ∗∗∗ for *p* < 0.001.

Next, decisions were made significantly faster in the low-demand than in the high-demand conditions (mean 5.85 ± 1.84 s vs. 7.38 ± 2.44 s; F[1, 16] = 28.28, *p* < 0.001, η^2^*p* = 0.64). As expected, larger choice sets took longer overall to decide upon (F[3, 48] = 61.33, *p* < 0.001, η^2^*p* = 0.79). There was also a significant demand × set size interaction (F[3, 48] = 10.59, *p* < 0.001, η^2^*p* = 0.40), reflecting that decision time increased more steeply with set size in the high-demand condition (Figure 2A, right). In essence, making the choice set easier with high-attractiveness options sped up decisions, but at the cost of accuracy.

Eye-gaze analysis confirmed that participants invested less visual search effort in the low-demand condition. Across all three gaze metrics, values were significantly lower with low demand than with high demand (screening ratio: F[1, 16] = 30.22, *p* < 0.001, η^2^*p* = 0.65; revisit ratio: F[1, 16] = 24.55, *p* < 0.001, η^2^*p* = 0.61; dwell bias: F[1, 16] = 18.61, *p* < 0.001, η^2^*p* = 0.54) (Figure 2B, left). As set size increased, the screening ratio dropped markedly (F[3, 48] = 190.05, *p* < 0.001, η^2^*p* = 0.92), and so did the revisit ratio (F[3, 48] = 16.47, *p* < 0.001, η^2^*p* = 0.51). It indicated that with more options, participants fixated on a smaller fraction of the items and made fewer revisits to them. In contrast, dwell bias did not differ by set size (F[3, 48] = 0.26, *p* = 0.854, η^2^*p* = 0.016). There was a strong demand × set size interaction for the screening ratio (F[3, 48] = 20.88, *p* < 0.001, η^2^*p* = 0.57): as the sets grew larger, participants inspected an even smaller proportion of options under the low-demand compared to the high-demand condition. Dwell bias also exhibited demand × set size interaction: it became more negative in the low-demand condition as set size increased (F[3, 48] = 4.85, *p* = 0.005, η^2^*p* = 0.23). Since a more negative dwell bias indicates a more uneven allocation of gaze on chosen options, this pattern suggests that in easy-choice scenarios, participants zeroed in on a few promising options. There was no significant demand × set size interaction for the revisit ratio (F[3, 48] = 2.55, *p* = 0.067, η^2^*p* = 0.14) (Figure 2B, right). Notably, aggregated gaze heatmaps revealed no systematic spatial bias (i.e., participants did not consistently favor any particular screen region), confirming that differences in search behavior were due to experimental manipulations rather than layout (Figure S2; representative scanpaths in Figure S5). A similar even distribution of gaze was observed in E2 (Figure S2).

Neural measures also revealed a pattern of reduced effort per item in the low-demand condition. The frontal N2 latency peaked significantly earlier in the low-demand than in the high-demand conditions (F[1, 16] = 6.67, *p* = 0.020, η^2^*p* = 0.29), suggesting that decision-related conflict was resolved more rapidly for each item when the options were easier (Figure 2C, left). Similarly, the parietal N1 and occipital P2 latencies were significantly shorter in the low-demand condition (F[1, 16] = 6.66, *p* = 0.020, η^2^*p* = 0.29; F[1, 16] = 5.17, *p* = 0.037, η^2^*p* = 0.24, respectively), indicating faster attentional orienting and perceptual-evaluative processing for each option when all options were highly attractive. Importantly, these demand-related differences varied with set size. As set size increased, all three FRP component latencies increased significantly (frontal N2: F[3, 48] = 23.06, *p* < 0.001, η^2^*p* = 0.59; parietal N1: F[3, 48] = 13.45, *p* < 0.001, η^2^*p* = 0.46; occipital P2: F[3, 48] = 63.26, *p* < 0.001, η^2^*p* = 0.80) (Figure 2C, right). Significant demand × set size interactions were observed for all three FRP latencies (frontal N2: F[3, 48] = 3.42, *p* = 0.024, η^2^*p* = 0.18; parietal N1: F[3, 48] = 2.94, *p* = 0.042, η^2^*p* = 0.16; occipital P2: F[3, 48] = 3.8, *p* = 0.016, η^2^*p* = 0.19), indicating that the divergence between low- and high-demand conditions became more pronounced as the number of options increased.

Likewise, each item fixation induced weaker occipital alpha suppression in the low-demand condition (F[1, 16] = 4.91, *p* = 0.042, η^2^*p* = 0.23) (Figure 2D, left), indicating that participants allocated less attentional effort to each option when all choices were appealing.^36^^,^^37^^,^^38^^,^^39^^,^^40^ Occipital alpha suppression also increased with set size (F[3, 48] = 13.7, *p* < 0.001, η^2^*p* = 0.46), reflecting higher attentional load as the assortment grew (Figure 2D, right). In contrast, the demand × set size interaction was not significant (F[3, 48] = 1.68, *p* = 0.184, η^2^*p* = 0.10), indicating that the demand-related reduction in attentional engagement was similar across set sizes. In sum, although increasing set size imposed growing neural costs per item, participants in the low-demand condition consistently processed each option with reduced cognitive effort, which became increasingly pronounced as the number of options grew.

After each choice, participants provided subjective ratings of their experience (Table 1). Paradoxically, even though the low-demand condition was rated as more enjoyable and more satisfying than the high-demand condition (F[1, 16] = 33.25, *p* < 0.001, η^2^*p* = 0.68; F[1, 16] = 40.2, *p* < 0.001, η^2^*p* = 0.72, respectively), participants reported lower confidence and higher regret in the low-demand condition (F[1, 16] = 22.01, *p* < 0.001, η^2^*p* = 0.58; F[1, 16] = 42.68, *p* < 0.001, η^2^*p* = 0.73, respectively). Set size had negligible effects on these self-reported measures (no consistent overload trend). Given the well-known limitations of post-choice self-reports, these subjective outcomes should be interpreted with caution.^41^^,^^42^^,^^43^

**Table 1:** Self-reported decision experience ratings in E1

Measure	High demand (mean ± SD)	Low demand (mean ± SD)	Condition F (η^2^_p_) *p* value	Set size F (η^2^_p_) *p* value	Interaction F (η^2^_p_) *p* value
Enjoyment	2.88 ± 1.24	5.36 ± 1.43	33.25 (0.68) <0.001 ∗∗∗	4.65 (0.23) 0.006 ∗∗	0.52 (0.031) 0.67 n.s.
Satisfaction	3.97 ± 1.47	5.46 ± 1.27	40.20 (0.72) <0.001 ∗∗∗	1.52 (0.087) 0.22 n.s.	0.85 (0.050) 0.48 n.s.
Confidence	3.25 ± 0.99	2.01 ± 0.87	22.01 (0.58) <0.001 ∗∗∗	0.37 (0.023) 0.78 n.s.	0.34 (0.021) 0.80 n.s.
Regret	2.92 ± 1.07	5.69 ± 1.20	42.68 (0.73) <0.001 ∗∗∗	5.97 (0.27) 0.002 ∗∗	0.15 (0.009) 0.93 n.s.

Statistical significance from two-way repeated-measures ANOVA is indicated as: n.s. for non-significant, ∗ for *p* < 0.05, ∗∗ for *p* < 0.01, and ∗∗∗ for *p* < 0.001.

### Experiment 2 (sequential narrowing)

In E2, using a bookmark-based decision aid to pre-filter options made choices significantly faster on average (F[1, 27] = 40.24, *p* < 0.001, η^2^*p* = 0.60), but did not improve choice performance. Overall, there were robust main effects of set size on choice performance (F[3, 81] = 33.1, *p* < 0.001, η^2^*p* = 0.55) and decision time (F[3, 81] = 63.02, *p* < 0.001, η^2^*p* = 0.70), reflecting a general decline in performance and increase in deliberation time as the number of options increased across both demand conditions. Yet, there was no main effect of demand on the rating difference (F[1, 27] = 0.21, *p* = 0.650, η^2^*p* = 0.010), nor any demand × set size interaction (F[3,81] = 0.060, *p* = 0.981, η^2^*p* = 0.002) (Figure 3A, left). Unlike in E1, the demand × set size interaction for decision time was not significant (F[3, 81] = 1.90, *p* = 0.136, η^2^*p* = 0.066), indicating that the aid sped up decisions by a similar amount regardless of set size (Figure 3A, right). Thus, even with the aid, participants still took longer to choose when more options were on the screen, scaling their deliberation time with the number of items.

**Figure 3:**
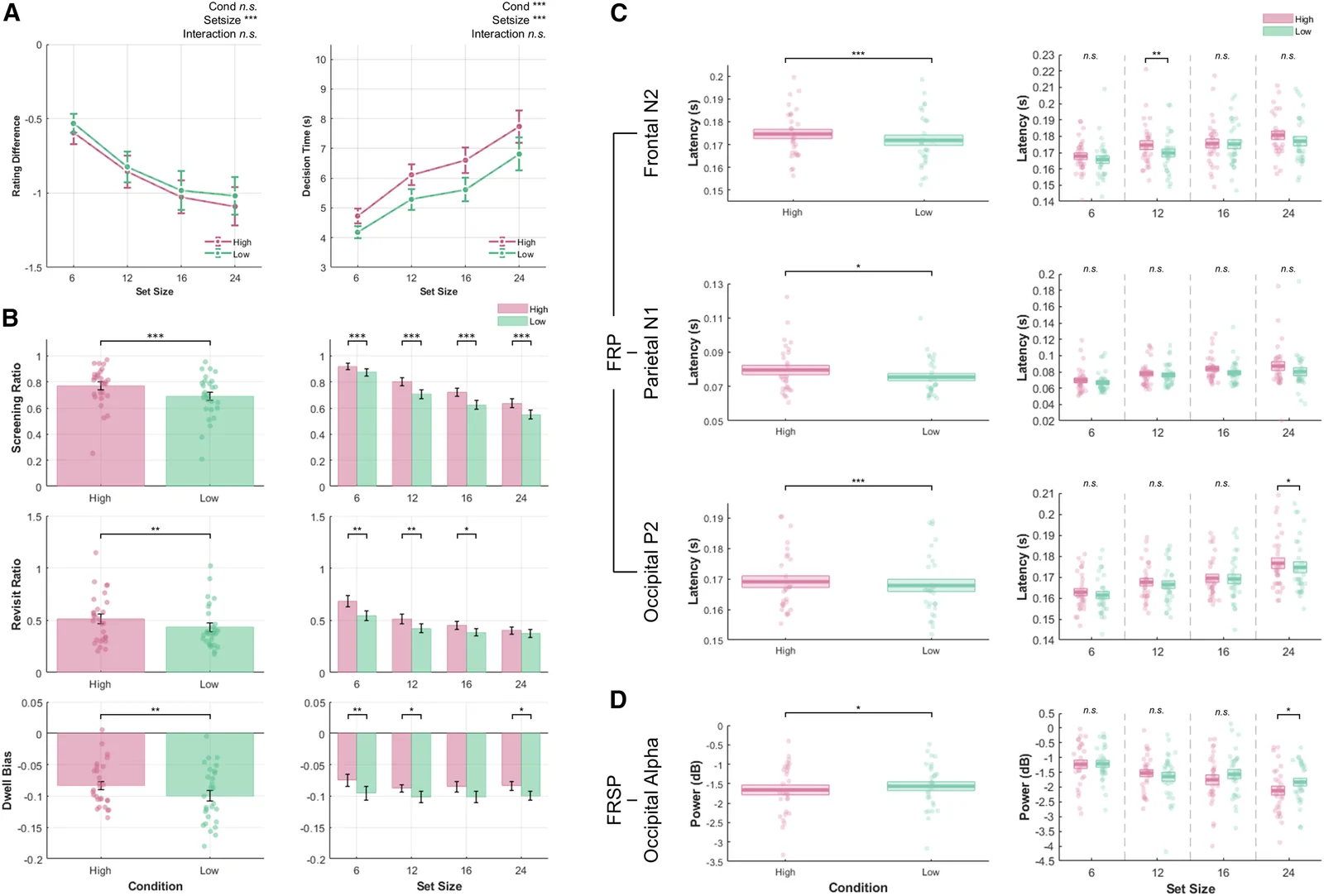
Results of E2 (sequential narrowing) (A) Rating difference between the chosen item and the best-rated item in the set (left) and decision time (right), shown by demand condition (high vs. low) and set size. (B) Eye-tracking measures—screening ratio, revisit ratio, and dwell bias—shown by demand condition (left; individual participants, *n* = 28) and by set size (right). (C) EEG FRP peak latencies. (D) EEG FRSP in the alpha band. In (A and B), error bars denote ±SEM. In (C and D) individual participants are shown (*n* = 28), and boxplots summarize the mean (center line) with boundaries indicating ±SEM. Statistical significance from two-way repeated-measures ANOVA: n.s., non-significant; ∗*p* < 0.05, ∗∗*p* < 0.01, ∗∗∗*p* < 0.001.

The eye-gaze measures in E2 mirrored those in E1. With the aid, participants fixated a smaller proportion of the options and made fewer back-and-forth comparisons than without the aid (screening ratio: F[1, 27] = 62.13, *p* < 0.001, η^2^*p* = 0.70; revisit ratio: F[1, 27] = 13.25, *p* = 0.001, η^2^*p* = 0.33; dwell bias: F[1, 27] = 11.73, *p* = 0.002, η^2^*p* = 0.30) (Figure 3B, left). Increasing the number of options again reduced the screening ratio and revisit ratio (F[3, 81] = 200.34, *p* < 0.001, η^2^*p* = 0.88; F[3, 81] = 73.65, *p* < 0.001, η^2^*p* = 0.73, respectively), while dwell bias showed no change with set size (F[3, 81] = 1.17, *p* = 0.326, η^2^*p* = 0.041). These effects were further qualified by significant demand × set size interactions for the screening ratio (F[3, 81] = 7.6, *p* < 0.001, η^2^*p* = 0.22) and revisit ratio (F[3, 81] = 4.07, *p* = 0.010, η^2^*p* = 0.13), indicating that as set size increased, the reduction in option coverage and comparative revisits under the aided condition became more pronounced. In contrast, the demand × set size interaction was not significant for dwell bias (F[3, 81] = 0.25, *p* = 0.861, η^2^*p* = 0.010). Together, these results indicate that the bookmarking step streamlined the decision process: with a self-curated shortlist, participants scanned options more efficiently and engaged in less back-and-forth deliberation (Figure 3B, right).

The EEG results revealed parallel effects of demand reduction on processing effort. As set size increased, processing of each option slowed overall: the frontal N2 latency became significantly longer with more options (F[3, 81] = 15.88, *p* < 0.001, η^2^*p* = 0.37), as did the parietal N1 and occipital P2 latencies (F[3, 81] = 7.29, *p* < 0.001, η^2^*p* = 0.21; F[3, 81] = 65.02, *p* < 0.001, η^2^*p* = 0.71, respectively). Demand condition also influenced these neural metrics. The frontal N2 peaked significantly earlier with the aid (F[1, 27] = 13.72, *p* < 0.001, η^2^*p* = 0.34), indicating that decision-related neural processing for each item was completed more quickly with the aid (Figure 3C, left). The parietal N1 and occipital P2 latencies were likewise shorter with the aid (F[1, 27] = 5.63, *p* = 0.025, η^2^*p* = 0.17; F[1, 27] = 14.44, *p* < 0.001, η^2^*p* = 0.35, respectively). In addition, each item fixation induced significantly less occipital alpha suppression when choices were pre-filtered (F[1, 27] = 4.51, *p* = 0.043, η^2^*p* = 0.14), reflecting reduced attentional load per option in the aided condition (Figure 3D, left). Unlike in E1, however, demand × set size interactions were not significant for any of the neural measures (all *p*s > 0.05), indicating that the effects of demand reduction on item-level processing speed and attentional engagement were similar across set sizes. Thus, although increasing set size similarly imposed growing neural costs per item, participants consistently responded to the decision aid by reducing effort per option across all set sizes, rather than reallocating their freed cognitive capacity to more detailed processing.

Subjective ratings in E2 mirrored those from E1 (Table 2). The low-demand condition was rated more enjoyable and more satisfying than the high-demand condition (F[1, 27] = 48.39, *p* < 0.001, η^2^*p* = 0.64; F[1, 27] = 38.91, *p* < 0.001, η^2^*p* = 0.59, respectively), but participants also reported lower confidence and higher regret (F[1, 27] = 21.48, *p* < 0.001, η^2^*p* = 0.44; F[1, 27] = 47.77, *p* < 0.001, η^2^*p* = 0.64, respectively). As in E1, set-size had minimal influence on these self-reports.

**Table 2:** Self-reported decision experience ratings in E2

Measure	High demand (mean ± SD)	Low demand (mean ± SD)	Condition F (η^2^_p_) *p* value	Set size F (η^2^_p_) *p* value	Interaction F (η^2^_p_) *p* value
Enjoyment	3.75 ± 1.25	4.48 ± 1.20	48.39 (0.64) <0.001 ∗∗∗	11.13 (0.29) <0.001 ∗∗∗	1.04 (0.037) 0.38 n.s.
Satisfaction	4.32 ± 1.04	4.84 ± 1.02	38.91 (0.59) <0.001 ∗∗∗	2.23 (0.076) 0.091 n.s.	0.55 (0.020) 0.65 n.s.
Confidence	2.43 ± 0.92	2.00 ± 0.66	21.48 (0.44) <0.001 ∗∗∗	3.79 (0.12) 0.013 ∗	1.47 (0.051) 0.23 n.s.
Regret	4.44 ± 0.89	5.17 ± 0.78	47.77 (0.64) <0.001 ∗∗∗	24.74 (0.48) <0.001 ∗∗∗	0.36 (0.013) 0.78 n.s.

Statistical significance from two-way repeated-measures ANOVA is indicated as: n.s. for non-significant, ∗ for *p* < 0.05, ∗∗ for *p* < 0.01, and ∗∗∗ for *p* < 0.001.

The following analyses were conducted separately in each experiment and are reported together to show that the pattern holds consistently across two independent samples. They ask whether demand modulates neural response magnitude as well as timing, add a complementary spectral marker (frontal-midline theta), examine the coupling among FRP latencies, and test the coherence of the behavioral, eye-gaze, and neural effort measures within individuals.

### Demand modulates processing speed, not response magnitude

To clarify what the FRP effects reflect, we re-analyzed the same components in the amplitude domain. Unlike the latency effects reported above, peak amplitudes of the parietal N1, occipital P2, and frontal N2 did not differ by demand in either experiment (smallest *p* = 0.198; Figures 4 and 5), and showed set-size effects only on the N1 amplitude (F[3, 48] = 3.00, *p* = 0.040, η^2^*p* = 0.16 in E1 and F[3, 81] = 7.48, *p* < 0.001, η^2^*p* = 0.22 in E2). The selective modulation of latency, but not amplitude, indicates that demand reduction changes how quickly each option is processed rather than the gross magnitude of the evoked response, consistent with a processing-speed (effort) interpretation.

**Figure 4:**
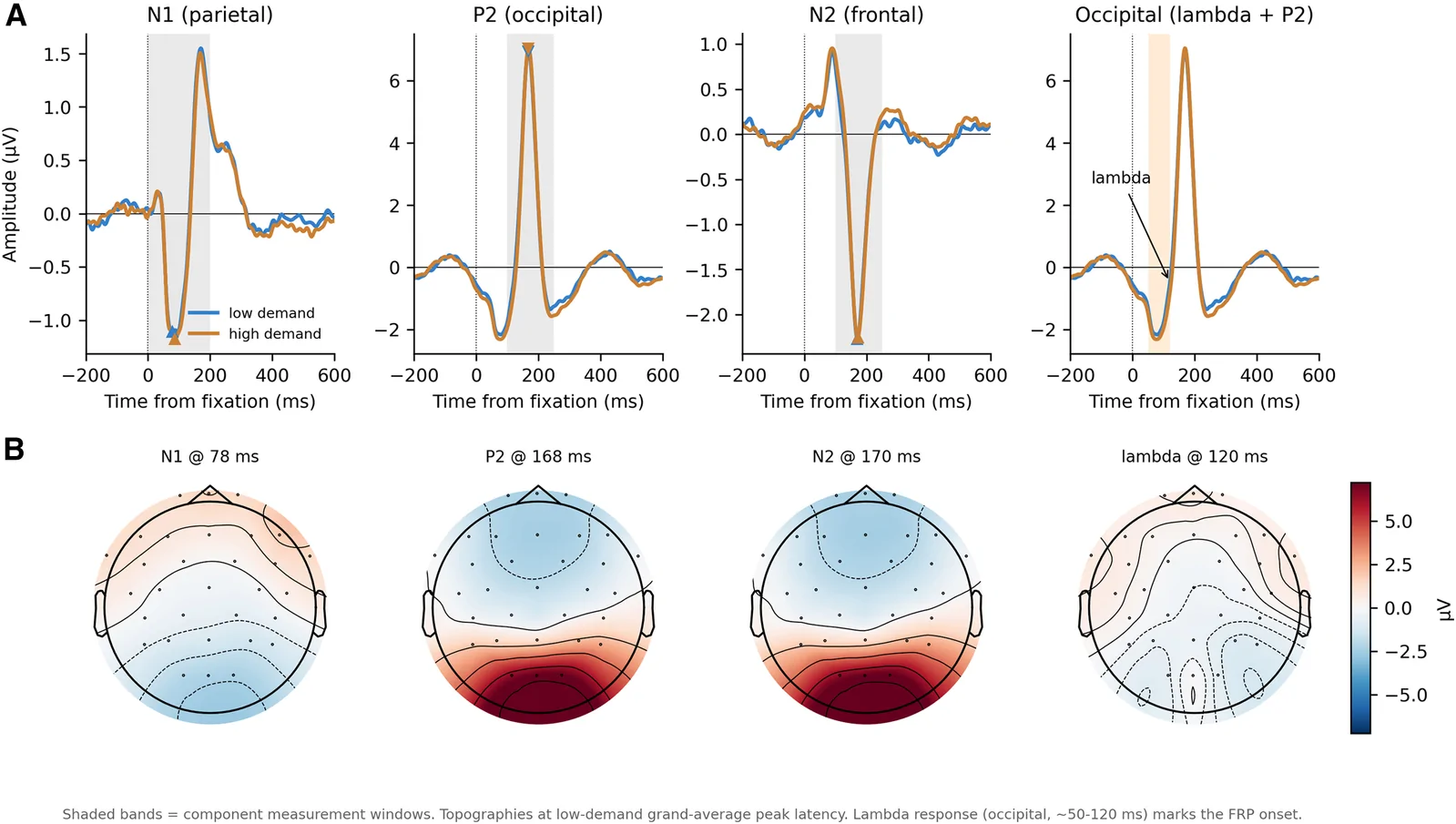
Grand-average FRPs and scalp topographies, E1 (related to Figure 2) (A) Grand-average FRP waveforms (*n* = 17) at the parietal N1, occipital P2, and frontal N2 clusters, plus the occipital channel showing the lambda response, by demand condition (low vs. high); shaded bands denote the component measurement windows and triangles mark the grand-average peaks. (B) Scalp topographies of the voltage distribution at each component’s grand-average peak latency. The occipital lambda response (∼80–120 ms) confirms the fixation-locked nature of the epochs; demand modulated component latencies (Figure 2C) but not amplitudes. Waveforms are grand averages across participants; no error bands are shown.

**Figure 5:**
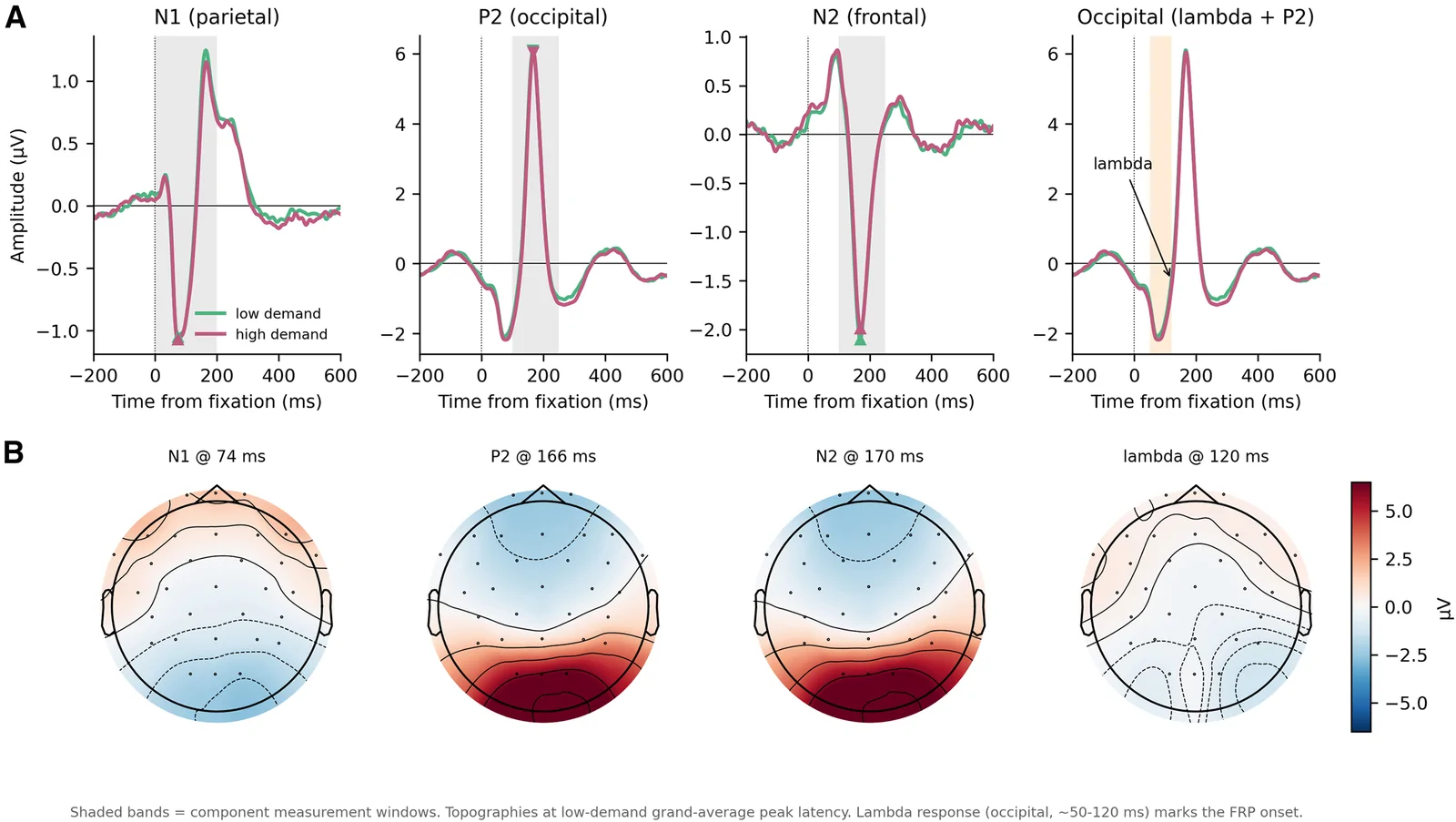
Grand-average FRPs and scalp topographies, E2 (related to Figure 3) (A) Grand-average FRP waveforms (*n* = 28) at the parietal N1, occipital P2, and frontal N2 clusters, plus the occipital channel showing the lambda response, by demand condition (low vs. high); shaded bands denote the component measurement windows and triangles mark the grand-average peaks. (B) Scalp topographies of the voltage distribution at each component’s grand-average peak latency. Demand modulated component latencies (Figure 3C) but not amplitudes. Waveforms are grand averages across participants; no error bands are shown.

### Frontal-midline theta tracks demand and set size

Extending the analysis beyond the alpha band, fixation-locked frontal-midline theta (4–8 Hz) power increased with demand in both experiments (E1 F[1, 16] = 7.67, *p* = 0.014, η^2^*p* = 0.32, and E2 F[1, 27] = 20.94, *p* < 0.001, η^2^*p* = 0.44) and rose with set size (E1 F[3, 48] = 13.56, *p* < 0.001, η^2^*p* = 0.46, and E2 F[3, 81] = 23.56, *p* < 0.001, η^2^*p* = 0.47, Figure S3). Because frontal-midline theta indexes cognitive control and conflict, its enhancement under high demand and large set sizes converges with the N2-latency findings and provides an independent spectral signature of the greater control demands that participants met with more effort only when demand was high.

### Component latencies are partly coupled across participants

To test whether the demand- and set-size-related latency shifts reflect a single general slowing or component-specific timing, we correlated participants’ mean component latencies (Figures 6A and 6B). The mid-latency occipital P2 and frontal N2 latencies were strongly correlated (r = 0.96 in E1 and r = 0.89 in E2), whereas the early parietal N1 was only moderately related to them (N1–P2 r = 0.49 and 0.64, N1–N2 r = 0.43 and 0.54, for E1 and E2, respectively). This pattern indicates a substantial shared timing component across the mid-latency window—partly attributable to the overlapping 100–250 ms measurement windows and to a general processing-speed factor—alongside a partial dissociation of the earliest attentional-orienting stage. Thus the demand effect is neither perfectly component-specific nor a single global shift.

**Figure 6:**
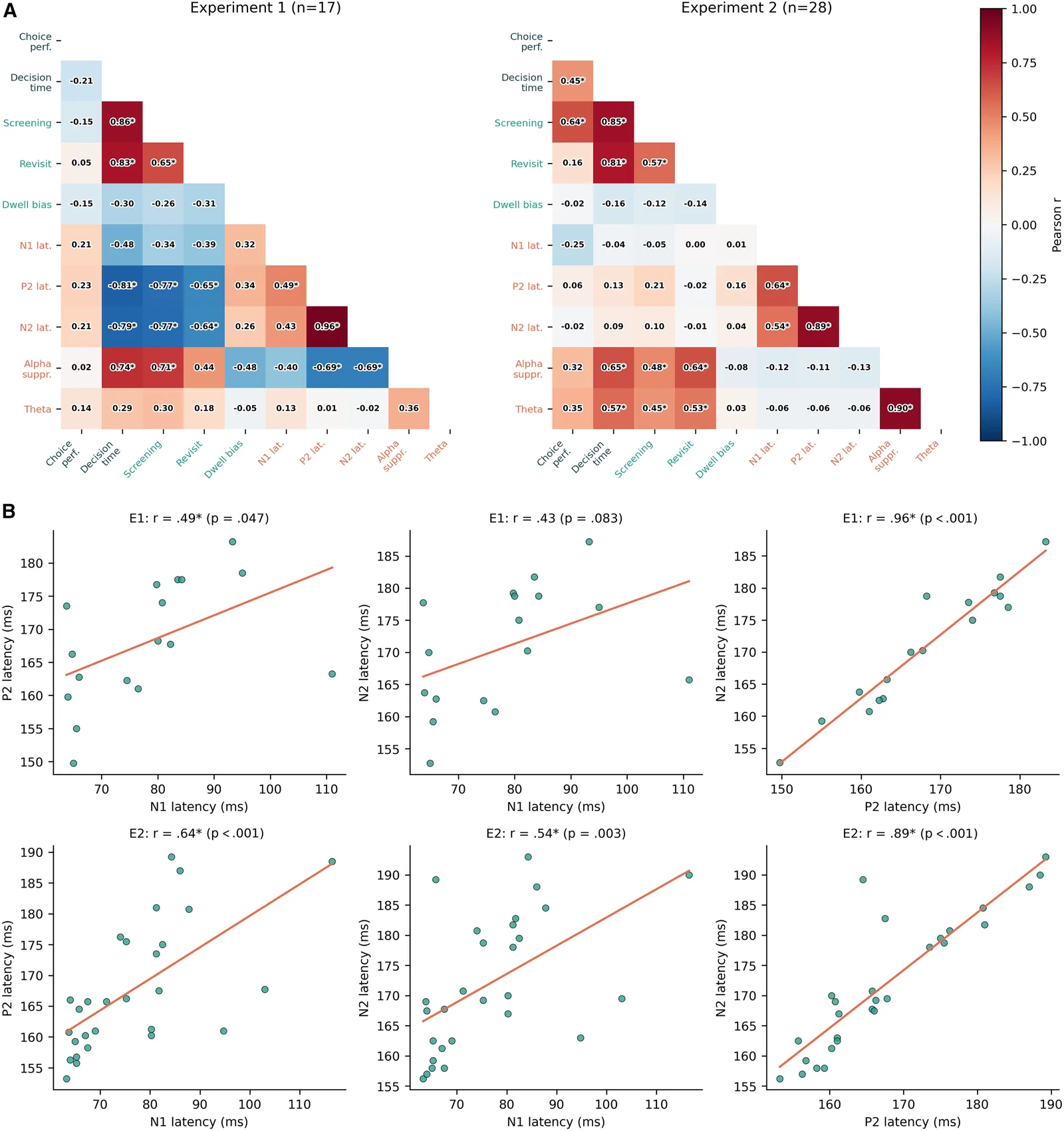
Cross-modal coupling of effort measures and FRP latency intercorrelations (related to Figures 2 and 3) (A) Cross-modal correlation matrices (Pearson r across participants) among behavioral (navy), eye-tracking (teal), and EEG (orange) measures for E1 (*n* = 17) and E2 (*n* = 28); ∗*p* < 0.05, two-tailed test of the Pearson correlation coefficient (uncorrected). Decision time, search breadth, and alpha suppression co-vary across individuals, indicating a shared effort dimension across modalities. (B) Per-participant FRP component-latency intercorrelations (N1–P2, N1–N2, P2–N2): mid-latency P2 and N2 latencies are strongly coupled, whereas the early N1 partly dissociates. Asterisks as in (A).

### Effort measures cohere across modalities

If behavioral, eye-gaze, and neural measures index a common underlying effort dimension, they should co-vary across individuals; they did. Participants who took longer to decide also searched more broadly (decision time × screening ratio, r = 0.86 in E1 and r = 0.85 in E2) and showed stronger occipital alpha suppression (decision time × alpha, r = 0.74 in E1 and r = 0.65 in E2), and broader search likewise tracked stronger alpha suppression (screening × alpha, r = 0.71 in E1 and r = 0.48 in E2; Figure 6A). This convergence across the behavioral, eye-gaze, and neural domains indicates that they capture a shared effort dimension rather than unrelated epiphenomena, and shows that the fixation-locked EEG adds explanatory value beyond behavior, since occipital alpha suppression indexes the depth of per-item engagement that gaze breadth—the screening ratio, i.e., the proportion of options fixated at least once—alone does not resolve.

## Discussion

Our results reveal why two widely deployed demand-reduction strategies—personalized filtering and sequential shortlisting—fail to mitigate choice overload despite making decisions feel easier. Across two experiments with converging behavioral, eye-gaze, and neural evidence, lowering external cognitive demand led participants to reduce internal effort rather than reallocate it toward more thorough evaluation. EEG and eye-tracking data pinpointed the mechanism: participants processed each option less deeply (shorter FRP latencies, weaker alpha suppression) and searched less broadly (lower screening ratios, fewer revisits), indicating effort conservation at both the item and set level. As a result, choice overload effects on objective performance persisted in the low-demand conditions, even though participants subjectively felt less overloaded.

In E1, presenting a set of all highly attractive options did not prevent the decline in choice performance as set size increased. Instead, participants showed a strong overload pattern in the low-demand condition, and neurophysiological data indicated why: they simply did not invest as much effort per item. Similarly, in E2, using a bookmark-based shortlist made the choice objectively easier, yet participants often completed the final decision with minimal effort, expending even less effort per option than they did without the aid. Consequently, they sometimes missed the optimal choice among those favorites. Thus, lowering external demands did not automatically translate into better decision performance, because people adjusted their effort downward and still ended up with suboptimal results.

### Choice overload persists if effort is under-allocated

This pattern—where reducing external cognitive demand led participants to disengage and conserve effort, while choice performance nonetheless continued to deteriorate as set size increased—supports a nuanced view of choice overload. Even though our interventions removed much of the objective difficulty, overload effects in performance (choosing inferior options as set size grows) were not eliminated. The eye-gaze data vividly illustrated this under-engagement: as more options were available, participants’ visual scanning became more fragmented and cursory. They spent less time on each item and skipped more items entirely as set size increased, indicating that it was hard to systematically evaluate a large set—presumably because they either could not attend to everything or assumed diminishing returns from doing so.

Additionally, our EEG results showed that each item in a larger set evoked stronger effort-related brain responses on average—implying each item was more mentally taxing to process in the context of a big choice set, likely because there were more comparisons in mind and more potential conflict to resolve. Ultimately, no matter how user-friendly one makes the choice environment, having to pick one option out of many imposes a cognitive load that can degrade performance. The bottleneck can be either an inability to evaluate everything (a capacity overload) or an unwillingness to do so (strategic under-effort), but both lead to the same outcome: not choosing the global optimum. This distinction has direct design implications: if the bottleneck is volitional rather than computational, merely reducing task difficulty will not suffice, and choice architectures must also sustain engagement.

In our experiments, it appears the dominant factor was the latter (“won’t”). Participants likely could have scrutinized more options or deliberated longer, but they did not because of effort-reward considerations. Our findings align with the idea that people adapt by conserving effort unless they perceive a clear benefit to expending more.^21^^,^^23^^,^^44^^,^^45^

### Effort conservation versus maximization

Our results emphasize that people do not automatically use all available cognitive resources to maximize decision accuracy. Consistent with prior work, individuals often avoid cognitively demanding processing even when doing so carries a performance cost.^23^ This paradox—where interventions intended to improve performance instead impair it through altered effort dynamics—extends beyond choice contexts. Even pharmaceutical cognitive enhancers have been shown to increase effort expenditure while decreasing its quality, ultimately harming task outcomes.^46^ Our findings add ecological validity to that notion—even in a rich, realistic decision like picking a movie for entertainment, people exhibit this effort aversion. This behavior is not mere laziness, but a reasonable adaptation from a cost-benefit standpoint. When the reward difference between options is small, additional cognitive effort yields little payoff, reducing willingness to engage.^47^ This dynamic was evident in E1. In the low-demand condition, where all options were highly attractive and little stood to be gained by identifying the single best one, participants exerted minimal effort. Only when options were less appealing (and thus, the “gem” carried a bigger payoff) did they ramp up effort. On this cost-benefit interpretation, what matters is the payoff for further effort, not how discriminable the options were. Within-set rating spread itself did not predict effort (see “Manipulation checks”), so the effect reflects how much was at stake, not how hard options were to tell apart.

Our observations coincide with prior research that decision-makers shift toward satisficing strategies when the task is easy, a pattern we observed not only with high-quality options (as in Huang and Xu^33^) but also with the use of a filtering tool, indicating this effort conservation occurs across both content- and process-based simplifications.^33^ These findings carry broad implications for consumer decision-making, recommender systems, and other applied choice environments: demand-reduction interventions—the dominant paradigm in modern choice architecture—may systematically underperform because they trigger the very disengagement they are designed to prevent.

### Converging behavioral, eye-gaze, and neural evidence

The same conclusion emerges independently at three levels of analysis, and these levels cohere within individuals.

Behaviorally, reducing demand consistently accelerated decisions without improving—and in E1 actively harming—choice quality as set size grew. This dissociation of speed from accuracy is the behavioral fingerprint of effort conservation: people banked the time saved rather than reinvesting it.

The eye-gaze data show how that time was saved. As choice difficulty decreased, attention became more selective rather than expansive—a pattern consistent with heuristic decision strategies. This kind of gaze behavior (skimming and re-checking only a limited set of favorites) is reminiscent of the “elimination by aspects” or other heuristic strategies described by Payne et al.,^48^ where decision-makers quickly eliminate many options based on key aspects and then compare just a small subset in depth.^48^ By contrast, participants’ broader search in the high-demand conditions suggests a more compensatory strategy. The eye-tracking results thus reveal a strategic shift from broad search under demand to a narrow focus when demand was eased.

At the neural level, the fixation-locked EEG showed that this strategic shift was accompanied by reduced per-item processing. The frontal N2 latency—an index of cognitive-control engagement—peaked later whenever participants faced situations demanding careful analysis (large sets or difficult choices), consistent with N2’s role in conflict monitoring and cognitive control^,^^49^ and frontal-midline theta power rose in parallel. Occipital alpha desynchronization, an index of visual attention and working-memory engagement, became stronger as more items entered the set, reflecting how visual working memory was taxed by tracking multiple items. In the low-demand conditions, weaker alpha suppression indicates that participants held less information in mind at once—considering fewer items concurrently or processing them less deeply. Critically, demand reduction shortened component latencies and weakened alpha suppression but left component amplitudes unchanged, and occipital alpha suppression co-varied with both decision time and search breadth across individuals (Figure 6). The fixation-locked EEG localizes the effect to the speed and depth of per-item processing, the stage that gaze breadth cannot resolve.

Across both experiments, subjective ratings revealed a consistent paradox. Low-demand conditions were perceived as more enjoyable and satisfying than high-demand conditions, yet they also elicited lower confidence and greater regret. This pattern supports prior findings that simplified decisions can undermine confidence, especially when the process feels too effortless.^35^^,^^50^ Participants may have second-guessed their choice due to reduced deliberation, whereas more difficult tasks, though less pleasant, fostered a sense of due diligence.^50^ Notably, these effects did not vary by set size, suggesting that perceived task difficulty and option quality, rather than assortment size, shaped reported confidence and satisfaction.

Our study thus contributes to the neuroscience of decision-making by linking these neural markers to the choice overload paradigm. Previous neuroimaging work (e.g., Reutskaja et al.^19^) showed that BOLD activation patterns change with choice set size; we extend those findings by showing how time-resolved brain activity at the level of individual fixations reflects the ongoing management of information.^19^ This approach enables a more fine-grained examination of decision processes over time, for example, by allowing inferences about when individuals disengage from further search or commit to a choice. More broadly, our multimodal framework—combining fixation-locked ERP latencies, oscillatory alpha and theta dynamics, and gaze-behavior metrics within a single paradigm—offers a template for studying effort allocation in other complex, multi-option decision environments.

### Implications for practical application

Overall, our study sheds light on the cognitive miser aspect of human decision-making in the context of choice overload. We demonstrate that reducing a task’s cognitive demand does not automatically translate into better objective decisions, because people often do not invest the extra effort they have been freed. Participants gravitated toward the path of least mental resistance, even when additional effort could have paid off. This highlights a crucial consideration for both theorists and practitioners: improving decision environments is only half the battle; the other half is ensuring that decision-makers engage with those improvements.

Our findings have implications for the design of recommender systems and other decision-support interfaces. Many online platforms use personalized filtering to mitigate choice overload, but our results suggest that simplification may also reduce users’ engagement with the decision. Accordingly, effective system design may require not only reducing the size of the choice set, but also sustaining deliberation among the remaining options. Indeed, a recent randomized controlled trial of personalized digital counseling demonstrated that when a recommendation system actively guided consumers through ranked alternatives rather than merely simplifying the choice set, participants discussed more options in depth and made substantially better decisions.^51^ Interface features that support this goal include tools for rapid side-by-side comparison of top candidates and lightweight prompts when a choice is made unusually quickly, which may signal insufficient evaluation of available options. This effort-conservation tendency also has consequences in high-stakes domains such as healthcare, finance, and public policy, where decision aids are increasingly used to simplify complex choices. Although these interventions can reduce overwhelm, they may also encourage minimal processing, so decision aids and policies may need safeguards that preserve engagement (e.g., highlighting meaningful within-set differences, adding brief accountability checks, or making key trade-offs salient). Overall, simplifying choices is most likely to improve outcomes when paired with mechanisms that maintain reflective evaluation.

### Limitations of the study

The two experiments were conducted with different participant samples, so direct comparisons between them should be made with caution. We designed each experiment to test a specific intervention separately, not to compare the two demand-reduction strategies head-to-head, and we therefore report them as independent studies rather than pooling them. The two samples did not differ on baseline task variables (number of eligible posters after familiarity screening, 189.0 vs. 185.2, *p* = 0.28; mean rating, 4.18 vs. 4.24, *p* = 0.88; rating dispersion, *p* = 0.31), so the convergent pattern across experiments is unlikely to stem from pre-existing differences between the two samples in these baseline task characteristics (stimulus familiarity and baseline rating behavior).

Several limitations qualify our conclusions. First, our samples consisted of young adults (mostly university students, aged 18–32), and we did not record participants’ occupations, domain expertise, or prior decision-making experience. The behavior of this relatively homogeneous group may not generalize to middle-aged or older adults, to other occupational or cultural groups, or to experienced decision-makers in a domain. For example, age-related changes in working memory could alter both the capacity for and the willingness to expend effort.^52^^,^^53^ Future work incorporating occupation, expertise, and a wider age range as measured variables would establish the boundary conditions of effort conservation.

Second, our task was low-stakes and hedonic—choosing a movie to watch—so the cost of a “wrong” choice is small. In high-stakes domains such as insurance, medical, or financial decisions, the cost of error is large and may sustain caution even when a platform reduces cognitive load, so effort conservation might be attenuated. Conversely, the same domains are where decision aids are most aggressively deployed. Whether effort conservation generalizes to high-stakes choices is an important open question.

Third, our task was administered in a controlled laboratory setting without the interactive interfaces, pop-ups, notifications, and external distractions of real apps and web pages. Whether effort conservation persists under such real-world interference remains to be tested in more naturalistic settings.

Finally, we did not explicitly manipulate or measure participants’ motivation to be accurate. We essentially assumed that everyone was trying their best within their comfort level, but it is possible that some participants simply did not care strongly about picking the top-rated movie (especially knowing it was just for a short trailer viewing). Manipulating incentives for accuracy would help separate “can’t” from “won’t” more directly.

## Resource availability

### Lead contact

Further information and requests for resources should be directed to and will be fulfilled by the lead contact, Sung-Phil Kim (spkim@unist.ac.kr).

### Materials availability

This study did not generate new unique reagents. The movie-poster stimulus set is available from the lead contact on request, subject to image-licensing constraints.

### Data and code availability

• De-identified behavioral, eye-tracking, and EEG data reported in this study have been deposited at OSF and are publicly available. The DOI is listed in the key resources table.
• All original code used to compute the reported measures, statistics, and figures has been deposited at OSF and is publicly available. The DOI is listed in the key resources table.
• Any additional information required to reanalyze the data reported in this study is available from the lead contact upon request.

## Acknowledgments

This work was supported by the 10.13039/501100003725National Research Foundation of Korea grant funded by the Korea government (MSIT) (RS-2025-00522357) and the 10.13039/501100010418IITP (Institute of Information & Communications Technology Planning & Evaluation) ITRC 10.13039/501100008122(Information Technology Research Center) grant funded by the Korea government (Ministry of Science and ICT) (IITP-2026-RS-2024-00437866).

## Author contributions

Conceptualization, S.S., S.L., N.L., and S.-P.K.; methodology, S.S., S.L., N.L., and S.-P.K.; investigation, S.S., S.L., N.L., and S.-P.K.; formal analysis, S.S. and S.L.; visualization, S.S.; supervision, S.-P.K.; funding acquisition, S.-P.K.; writing – original draft, S.S., S.L., and S.-P.K.; writing – review and editing, S.S., S.L., N.L., and S.-P.K.

## Declaration of interests

The authors declare no competing interests.

## STAR★Methods

### Key resources table

**Table:** 

REAGENT or RESOURCE	SOURCE	IDENTIFIER
**Deposited data**		

Behavioral, eye-tracking, and EEG-derived data (this paper)	This paper	OSF: https://doi.org/10.17605/OSF.IO/NXFS5

**Software and algorithms**		

MATLAB R2021a (or later)	MathWorks	RRID:SCR_001622
EEGLAB	Delorme and Makeig^54^	RRID:SCR_007292
ICLabel (EEGLAB plugin)	Pion-Tonachini et al.^55^	https://github.com/sccn/ICLabel
Artifact Subspace Reconstruction (clean_rawdata)	Chang et al.^56^	https://github.com/sccn/clean_rawdata
Python 3.12 (numpy, scipy, pandas, matplotlib, h5py)	Python Software Foundation	RRID:SCR_008394
Titta toolbox (Tobii control)	Niehorster et al.^57^	https://github.com/dcnieho/Titta
Custom analysis code (this paper)	This paper	OSF: https://doi.org/10.17605/OSF.IO/NXFS5

**Other**		

Tobii Pro TX300 eye-tracker (300 Hz)	Tobii AB	N/A (instrument)
actiCHamp 31-channel EEG system (500 Hz)	Brain Products GmbH	N/A (instrument)
200 movie-poster stimuli (700 × 1000 px)	This paper	Available from lead contact

### Experimental model and study participant details

#### Participants

Eighteen subjects (13 female; mean age 21.61 ± 2.81 years; age range: 18–27) participated in Experiment 1, and a separate group of thirty subjects (16 female; mean age 23.20 ± 3.97 years; age range: 19–32) participated in Experiment 2. After exclusions due to eye-data corruption, the data of seventeen and twenty-eight participants were analyzed in Experiments 1 and 2, respectively. Participants were predominantly university students. Occupation, domain expertise, and prior decision-making experience were not recorded (see limitations of the study). All participants had normal or corrected-to-normal vision and reported no history of neurological, psychiatric, or significant medical issues. They all provided written informed consent prior to the experiment in accordance with the Declaration of Helsinki and according to the approval obtained from the Institutional Review Board of the Ulsan National Institute of Science and Technology (UNISTIRB-21-35-C) and were paid for their participation after the experiment.

### Method details

#### Stimuli and materials

We used movie choice as the decision task because it is ecologically common, intrinsically motivating and hedonic,^5^ and elicits genuine idiosyncratic preferences that can be measured per item, which is essential for defining choice performance against each participant’s own ratings. The choice options in both experiments were drawn from a large pool of movie posters. We compiled a database of 200 movie options, each represented by its poster image (700 × 1000 px). The positions of the option images on screen were arranged within a fixed 3 × 8 grid and randomized on each trial. For trials with fewer than 24 options, only a subset of grid positions was populated, following predefined spatial patterns that distributed items evenly across the grid while leaving the remaining positions empty (representative layouts in Figure 1D). This ensured that smaller set sizes occupied the full spatial extent of the display rather than being clustered in a single region. All grids were centered on the display, and spacing between items was kept uniform. This layout strategy ensured no particular screen region was consistently advantaged or disadvantaged. Each movie poster image subtended ∼5.5 ° × 9.7 ° of visual angle, with ∼2.5° gaps between images. The screen background was uniform gray. Participants were instructed that on each trial, they should freely view the options and select the movie they would most like to watch (Figure 1).

#### Apparatus and software

Experimental sessions took place in a quiet, dark, electromagnetically shielded booth. Participants sat ∼60 cm from a 23″ LCD monitor (1920 × 1080 resolution, 60 Hz refresh). The eye-tracker (Tobii Pro TX300, Tobii AB, Sweden) was embedded below the monitor to record gaze at 300 Hz. A standard 9-point calibration was performed at the start and repeated as needed until the validation error was <0.5°. Head position was stabilized with an adjustable chin-and-forehead rest to maintain calibration accuracy. EEG was recorded concurrently using 31-channel wet electrodes (actiCHamp, Brain Products GmbH, Germany). Electrodes were placed according to the international 10–20 system, with additional ground (left mastoid) and reference (right mastoid) electrodes. Impedances were kept below 10 kΩ throughout the recording, and the signals were sampled at 500 Hz. The stimulus presentation and data synchronization were controlled by custom scripts in MATLAB (MathWorks, Natick, MA, USA). Precise timing of stimulus presentation updates and event markers was synchronized with EEG signals using the parallel port trigger output to the EEG system.

#### Experimental design

We conducted two separate but conceptually parallel experiments, each employing a 2 × 4 within-subjects factorial design. The factors were Demand (high-demand vs. low-demand) and Set Size (number of options: 6, 12, 16, or 24). The set sizes were chosen to span the range over which choice overload typically emerges, with 6 below and 12–24 at or above commonly reported overload thresholds.^10^^,^^19^^,^^34^ While 18 alternatives could be a plausible intermediate level between 12 and 24, we used a set size of 16 to match the consistency with the stimulus-generation method and counterbalancing constraints. Specifically, each demand condition drew from a fixed pool of 48 items – the least common multiple of the set sizes – allowing the stimulus-generation algorithm to partition the pool into balanced subsets for each set size and to populate a fixed 3 × 8 grid using predefined position templates with even spatial sampling. In both experiments, each participant completed choice trials under both demand conditions and all set sizes, allowing repeated-measures comparisons. Experiment 1 manipulated cognitive demand through the choice set content, whereas Experiment 2 manipulated demand through the decision process.

In Experiment 1, the low-demand condition presented choices from high-attractiveness sets (all options with high initial appeal), while the high-demand condition used low-attractiveness sets (options of lower appeal). The high-attractiveness pool comprised the 48 highest-rated posters based on each participant’s initial ratings (see below for the rating procedure), and we computed the mean and variance of ratings within this pool. The low-attractiveness pool was then selected from the remaining posters using an optimization procedure that searched candidate 48-item subsets and chose the subset that maximized the mean rating difference from the high-attractiveness pool while keeping the rating dispersion comparable. This ensured the two pools had similar rating variance while maximizing the overall rating difference.

In Experiment 2, participants were provided a bookmarking tool (decision aid) to narrow the options before the choice task. In the low-demand condition, the final choice set was constructed from a screened pool anchored on the participant’s bookmarked items, whereas in the high-demand condition, the final set was drawn from a control pool containing only non-bookmarked items. Because the number of bookmarks varied across participants, we utilized an algorithmic procedure to ensure that the screened and control pools were both fixed to 48 items and matched in rating distribution. Specifically, if a participant bookmarked more than 48 items, the screened pool was defined as the 48 highest-rated bookmarked items. If fewer than 48 bookmarked items were available, the screened pool was completed by adding non-bookmarked items selected from the highest available rating values until the pool reached 48 items (i.e., fillers were drawn preferentially from the top of the remaining rating-ranked non-bookmarked candidates; selection within a given rating value was randomized). On average, 18.93 bookmarked items (SD = 8.06) were included in the 48-item screened pool. To ensure that any differences between conditions reflected bookmarking rather than option quality, we matched the rating distribution of the screened and control pools. Specifically, after the screened pool was finalized, we then constructed the control pool by sampling exclusively from non-bookmarked items such that, for each rating bin, the control pool contained the same number of items as the screened pool. When multiple candidates were available within a bin, items were selected at random. Thus, the screened and control pools were identical in rating distribution and differed only in whether items had been bookmarked. Given the smallest set size of 6, we required that the screened pool contain at least 16.67% bookmarked items to ensure that each screened display could include at least one bookmarked option. Because 16.67% of 48 corresponds to 8 items, participants would have been excluded if fewer than 8 bookmarked items were available for the screened pool; no participants were excluded based on this criterion.

Within each experiment, trial conditions were counterbalanced and randomized. In both Experiment 1 and 2, all trials were intermixed in random order (since no additional instruction difference was needed between conditions). Each participant experienced every combination of Demand and Set Size an equal number of times. Specifically, each of the 8 conditions (2 demand × 4 set sizes) was presented 20 times, yielding 160 choice trials per participant in each experiment. Additionally, Forced Choice (FC) trials were included as attention-check control trials in which participants were instructed to choose the highlighted item regardless of preference, allowing us to verify attention and task compliance under identical visual displays. FC trials were presented 4 times for each of the 8 conditions (2 demand × 4 set sizes), yielding 32 FC trials and a total of 192 trials for all conditions per participant. The trials were separated into 4 blocks considering individuals’ fatigue. Each block consisted of the same number of trials for each condition, which would result in 48 trials (2 demand × 4 set sizes × 5 trials + 1 FC × 8 trials). Trial order within blocks was randomized for each participant. Within each trial, no item was shown more than once, and stimulus scheduling was used to distribute repeated appearances of posters approximately evenly across trials within each condition and set size, minimizing systematic familiarity differences between conditions.

#### Tasks

Each participant completed a single session (∼3 h including setup). The sequence of tasks was as follows (Figure 1A):

**Task 1: Familiarity Check.** Participants were shown 200 movie posters one by one and asked if they had watched the movie before. Responses were obtained in binary options (1 = I have watched the movie before, 0 = I have never watched the movie before). Only the ones they had not watched before were used for further tasks. On average, participants contributed 189.00 (SD = 10.64) eligible posters in Experiment 1 and 185.21 (SD = 11.55) eligible posters in Experiment 2 after this familiarity screening.

**Task 2: Rating Task.** Participants were shown the unwatched movies one by one again. This time, participants rated how much they wanted to watch the movie by clicking the rating bar below the poster image. An 11-point Likert scale was used for the rating bar (1 = I do not want to watch the movie at all, 11 = I want to watch the movie very much). All stimuli were presented in random order. After completing one round of the rating task, participants went through another identical round, also in random order, making a total of two rounds of the rating task. The mean rating of each stimulus was calculated by averaging the two ratings.

**Task 2**-**1: Bookmark Task.** This task was done only for Experiment 2. Participants went through a bookmark task right after the rating task. A maximum of 24 posters were shown on the screen while participants freely clicked on those they would like to bookmark. A heart-shaped icon appeared above the selected poster, indicating a bookmark. Multiple selection was allowed, and the task was self-paced until all unseen posters from Task 1 were presented.

**Task 3: Choice Task.** The objective of the choice task was to choose the most desirable option. Participants were informed that they would watch a short review of one of their chosen movies after the experiment. Each participant completed 4 blocks of 48 trials each with a short break between the blocks. Choices were self-paced with no explicit time limit; the trial ended when a selection was made.

**Task 4: Post-Choice Ratings.** After each choice trial in both experiments, participants answered a brief set of self-report questions about their decision experience (while the EEG/eye-tracking recording continued passively during this period). Specifically, they rated: (a) how enjoyable the decision process was, (b) how satisfied they were with their chosen option, (c) how confident they were that they made the best choice, and (d) how much regret they felt. Ratings were made on 7-point Likert scales presented on the screen (tailored to each question, e.g., 1 = not at all enjoyable, 7 = extremely enjoyable). Participants pressed number keys to submit their responses. Once the questions were completed, the next choice trial began.

Throughout the procedure, we monitored EEG and eye-tracking signals in real-time for quality. If drift or signal loss was detected (e.g., due to cap shift or calibration error), the experiment was paused to recalibrate the eye-tracker or adjust electrodes. Such pauses were infrequent, and data collection resumed once signals were stable.

#### Trial structure

Each choice trial followed a fixed timeline (Figure 1C). A central fixation cross appeared at trial start for 2 s, giving participants a moment to focus and blank out previous visual stimuli. The choice set then appeared (EEG trigger 32), and participants began exploring the options freely. In all conditions, the choice set display remained on-screen until a decision was made (self-paced). Participants indicated their choice by clicking their chosen item using a mouse (EEG trigger 30). The time from set onset to mouse click constituted the decision time measure for that trial. Once a choice was made, the screen cleared, and the four experience-rating questions (Task 4) were presented one by one at the center. Participants responded to each within a few seconds (self-paced), and this pacing ensured participants had a short break to relax their eyes between trials.

#### Eye-tracking acquisition and preprocessing

Eye-tracking data were processed to extract gaze fixation events and visual attention measures. A fixation was defined as a period during which gaze remained within an Area of Interest (AOI) for at least 100 ms. Brief data gaps (<100 ms) were bridged through linear interpolation. After detecting fixations, we mapped each fixation to a specific choice option or to “blank” (if the fixation fell on background areas). Only fixations on actual option items were retained for effort analyses. Each option’s on-screen image was defined as an AOI with a bounding box slightly larger than the image to capture near-boundary gaze points.

#### EEG acquisition and preprocessing

EEG data were preprocessed and analyzed using the EEGLAB toolbox and custom MATLAB scripts. Continuous EEG recordings were first high-pass filtered (0.1 Hz) to remove slow drifts. A notch filter at 60 Hz was applied to attenuate line noise. Then, a band-pass filter from 1 to 50 Hz was applied (finite impulse response filter, zero-phase). Bad channels were detected using kurtosis-based criteria and were interpolated using spherical spline interpolation (on average 1–2 channels per participant).^54^^,^^58^ After that, the signals were re-referenced by the common average reference (CAR) method. We performed an Independent Component Analysis (ICA) to identify and remove residual oculomotor artifacts. The data were then processed with an Artifact Subspace Reconstruction (ASR) method to remove high-amplitude transient artifacts. We used a conservative threshold of 20 standard deviations for the ASR cutoff, which has been shown to effectively suppress large artifacts while preserving genuine EEG signals.^56^ Next, we segmented the EEG into epochs time-locked to fixation onsets, as described below.

### Quantification and statistical analysis

#### Behavioral measures

The primary behavioral outcomes were defined at the trial level and summarized within each Demand × Set Size condition. Choice performance was the difference between the rating of the chosen item and the rating of the highest-rated item among the displayed options, based on each participant’s own pre-task ratings (values ≤0; closer to 0 = better). Decision time was the latency from choice-set onset to the response click. Post-choice experience ratings (enjoyment, satisfaction, confidence, regret) were taken from Task 4. Trial-wise measures were averaged within each condition for each participant.

#### Eye-gaze measures

From the sequence of fixations on each trial, we computed three eye-gaze metrics characterizing visual search behavior.^59^^,^^60^^,^^61^^,^^62^^,^^63^ The Screening Ratio was the proportion of available options fixated at least once (number of uniquely fixated options ÷ set size); a value of 1 means every option was looked at. The Revisit Ratio indexed comparative look-backs as the average number of returns to an option after it was first seen (total item fixations minus uniquely fixated items, divided by uniquely fixated items); 0 indicates a pure single-pass scan. The Dwell Bias quantified gaze concentration by comparing the proportion of total decision time spent on unchosen options with the proportion of fixated options that were unchosen; it is centered at 0, with negative values indicating that fixation time was concentrated on the chosen option rather than distributed across the unchosen options. Together these provide a profile from breadth of exploration to depth of comparative examination.

#### Fixation-related potential (FRP) measures

We extracted fixation-locked EEG epochs for each option fixation during the choice tasks. Using the eye-tracking data, we marked each instance when the participant fixated on an option (AOI) in the EEG. Epochs were defined from 200 ms before to 800 ms after fixation onset. Because successive fixation epochs overlap, all fixation epochs in a trial were baseline-corrected against a common pre-trial baseline (the mean of −200 to 0 ms relative to trial onset).^64^ A post-hoc baseline shift was applied for visualization only and did not affect any statistics.

Fixation-related potentials differ from stimulus-locked visual evoked potentials because each epoch is triggered by a self-generated saccade onto an option in free viewing. The characteristic FRP signature is the lambda response, an occipital positivity peaking ∼80–120 ms after fixation onset that reflects the visual inflow following the saccade.^64^^,^^65^^,^^66^ We confirmed a clear lambda response at occipital sites (Figures 4 and 5) and, following the FRP literature, used it to anchor interpretation of the later components. For our effort analyses we focused on three subsequent deflections, which are well characterized in the visual-ERP literature and which we measured here on the fixation-locked waveform: an early posterior N1 (attentional orienting),^67^ a mid-latency occipital P2 (perceptual/decision processing),^68^ and a later frontal N2 (conflict monitoring/cognitive control).^49^^,^^69^ The N1 was measured as the most negative deflection within 0–200 ms at a pooled parietal cluster (Pz/P3/P4); the P2 as the most positive deflection within 100–250 ms at an occipital cluster (Oz/O1/O2); and the N2 as the most negative deflection within 100–250 ms at a frontal cluster (Fz/F3/F4). For each participant and condition we extracted the peak latency of each component as an index of per-item processing speed (longer latency = more effortful processing).^65^ We additionally extracted the peak amplitude of each component to test whether demand modulated response magnitude as well as timing.

#### Fixation-related spectral perturbation (FRSP) measures

In addition to time-domain FRPs, we analyzed the EEG in the time-frequency domain using a wavelet-based decomposition of each fixation epoch. Each epoch’s power was normalized to the common pre-trial baseline and expressed in decibels. We focused on occipital alpha (8–13 Hz) activity (Oz/O1/O2), where greater suppression (desynchronization) indexes stronger attentional engagement^,^^36^^,^^37^^,^^38^^,^^39^^,^^40^ extracting the alpha desynchronization magnitude as the largest decrease in alpha power within 0–300 ms after fixation onset. To extend the spectral characterization, we additionally extracted fixation-locked frontal-midline theta (4–8 Hz) power at the frontal cluster, a marker of cognitive control and conflict.

#### Statistical analysis

For each experiment and each dependent measure, we fit a two-way within-subjects (repeated-measures) ANOVA with Demand (low vs. high) and Set Size (6, 12, 16, 24) as factors, implemented with MATLAB’s fitrm and ranova functions. An alpha level of 0.05 (two-tailed) was used. We report F-statistics with numerator/denominator degrees of freedom, exact *p*-values (reported as *p* < 0.001 when smaller), and partial eta squared (η^2^p) effect sizes. Post-hoc pairwise comparisons used paired t-tests with Bonferroni correction. In figures and tables, statistical significance from these tests is indicated as n.s. (not significant), ∗*p* < 0.05, ∗∗*p* < 0.01, and ∗∗∗*p* < 0.001.

FRP component amplitudes and frontal theta were analyzed with the same 2 × 4 RM-ANOVA as above.

##### Component-latency intercorrelations

To test whether latency effects reflect general slowing versus component-specific timing, we computed Pearson correlations across participants among the per-participant mean N1, P2, and N2 latencies.

##### Cross-modal correlations

To test whether behavioral, eye-gaze, and neural measures index a common effort construct, we computed Pearson correlations across participants among per-participant means of choice performance, decision time, screening ratio, revisit ratio, dwell bias, N1/P2/N2 latency, alpha suppression, and theta power (Figure 6).

##### Manipulation checks

Convergent validity of the demand manipulation was assessed with paired t-tests on decision time and screening ratio (low vs. high demand). To test whether within-set rating spread (rather than the intended manipulation) drove effort, we computed within-subject correlations (subject-mean-centered) between each free-choice trial’s within-set rating standard deviation and its decision time and number of fixations, pooled and within each demand condition.

##### Rating distributions

For each participant we summarized the distribution of pre-task poster ratings (mean, SD, range) to verify non-uniform rating behavior (Figure S4). Between-experiment comparability was assessed with independent-samples t-tests on per-participant number of eligible posters, mean rating, and rating SD.

These analyses were conducted in Python 3.12 (numpy, scipy, pandas). The repeated-measures ANOVA pipeline reproduced the F-statistics reported in the main text for all behavioral and eye-gaze measures, and all analysis code is provided in the deposited repository.

### Additional resources

This study did not generate additional resources.
