# Supplementary material for: Reducing cognitive demand fails to mitigate choice overload: Multimodal neural and gaze evidence for effort conservation
Source: iScience. 2026 Sep 24;29(10):117616. doi: 10.1016/j.isci.2026.117616 (PMC13634898; doi:10.1016/j.isci.2026.117616)
Supplement: Supplementary file 1 [file mmc1.pdf]

iScience, Volume *29*

# Supplemental information

# Reducing cognitive demand fails to mitigate choice overload: Multimodal neural and gaze evidence for effort conservation

**Seungbeom Seo, Seungji Lee, Nayoung Lee, and Sung-Phil Kim**

# Supplemental Information

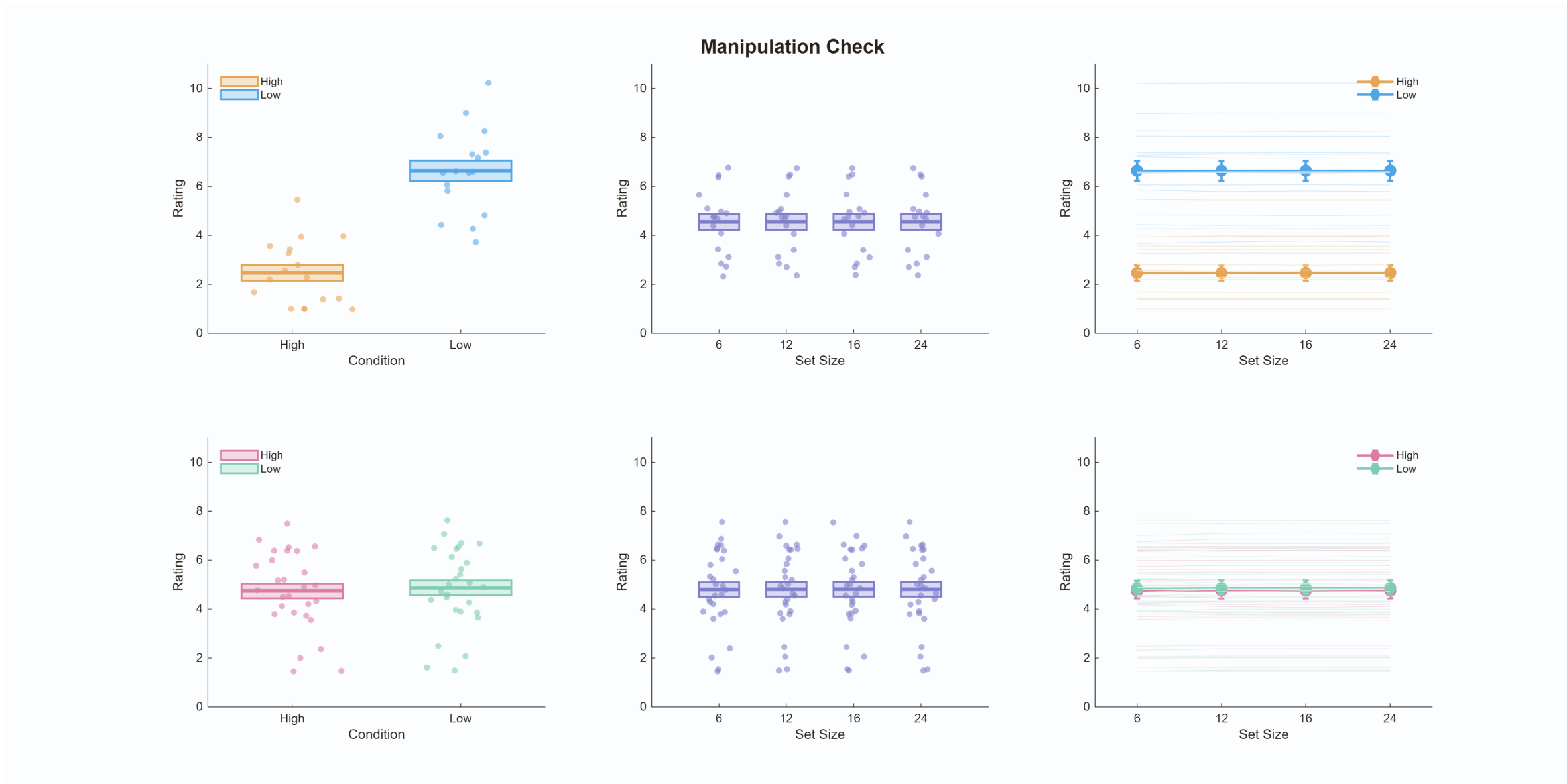


**Figure S1. Manipulation check, related to STAR Methods.**

Plots show the mean (±SEM) rating of the stimulus pools used to construct the choice sets under each demand condition for Experiments 1 and 2 (ratings obtained from each participant's initial rating task on an 11-point scale). In Experiment 1 (E1), the high-attractiveness (low-demand) pool was rated higher than the low-attractiveness (high-demand) pool, confirming the intended manipulation (two-way repeated-measures ANOVA: main effect of Demand, $F_{1,16} = 1129.6$, $p < 0.001$, $\eta^2_p = 0.89$). In Experiment 2 (E2), the screened (low-demand) and matched control (high-demand) pools were constructed to have closely similar attractiveness, so the mean difference between conditions was small ($\Delta = 0.13$ on the 11-point scale) even though the main effect of Demand was statistically significant ($F_{1,27} = 251.54$, $p < 0.001$), consistent with equating option quality across conditions while varying whether choice sets contained bookmarked items.

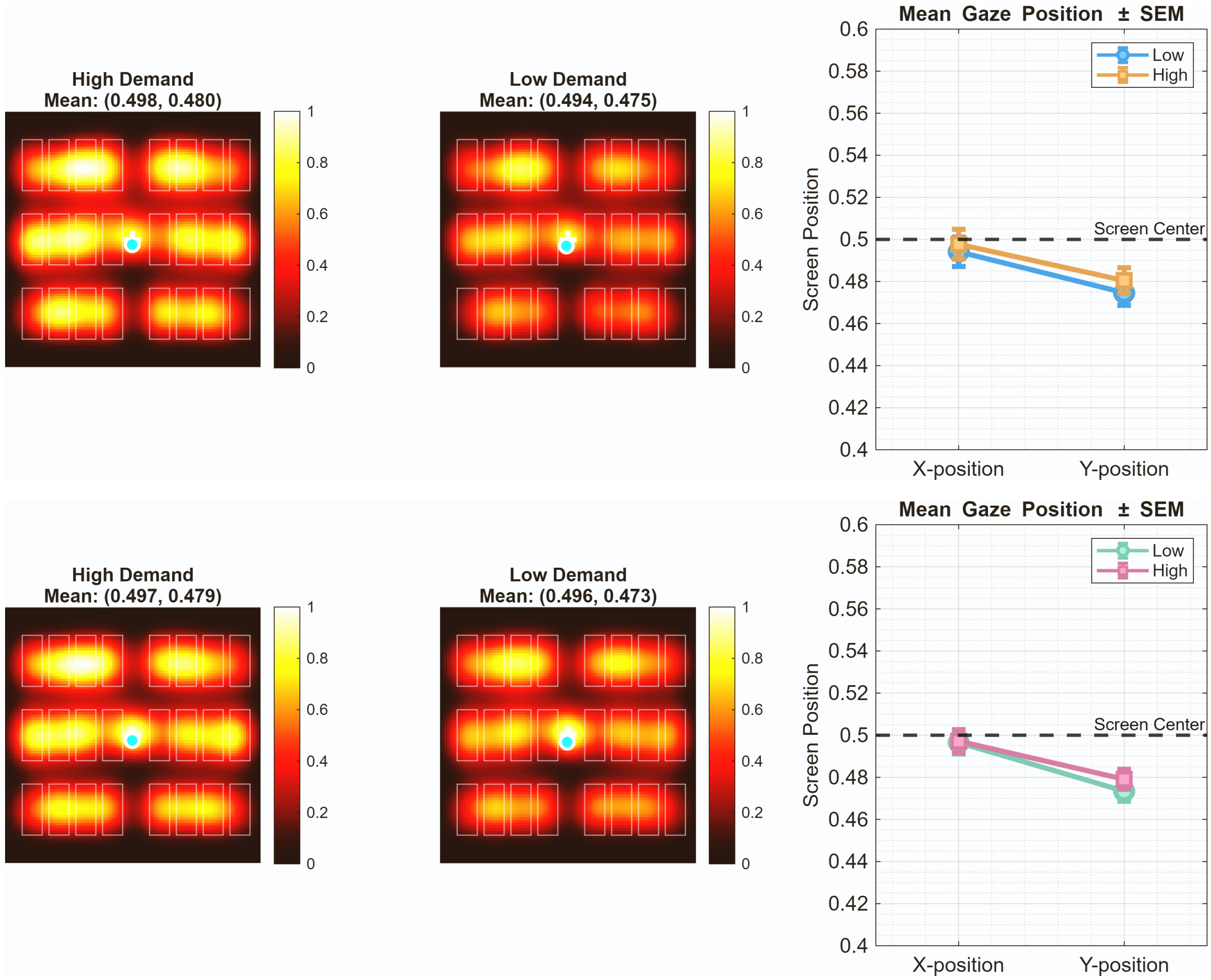


**Figure S2. Gaze heatmaps and mean gaze position show no systematic spatial viewing bias (Experiments 1 and 2), related to Figures 2 and 3.**

Aggregated gaze heatmaps for the high- and low-demand conditions with the mean gaze position overlaid (normalized screen coordinates), shown for both experiments. Gaze distributions were highly similar across conditions, with mean gaze positions clustered near screen center, and two-way repeated-measures ANOVAs on x- and y-position revealed no systematic spatial bias (Experiment 1: x, $F_{1,16} = 2.09$, $p = 0.17$; y, $F_{1,16} = 1.25$, $p = 0.28$. Experiment 2: x, $F_{1,27} = 0.04$, $p = 0.85$; y, $F_{1,27} = 1.33$, $p = 0.26$). Outlined boxes mark the fixed 3 × 8 poster slots of the choice array, shown so the gaze density can be read against the display the participants saw.

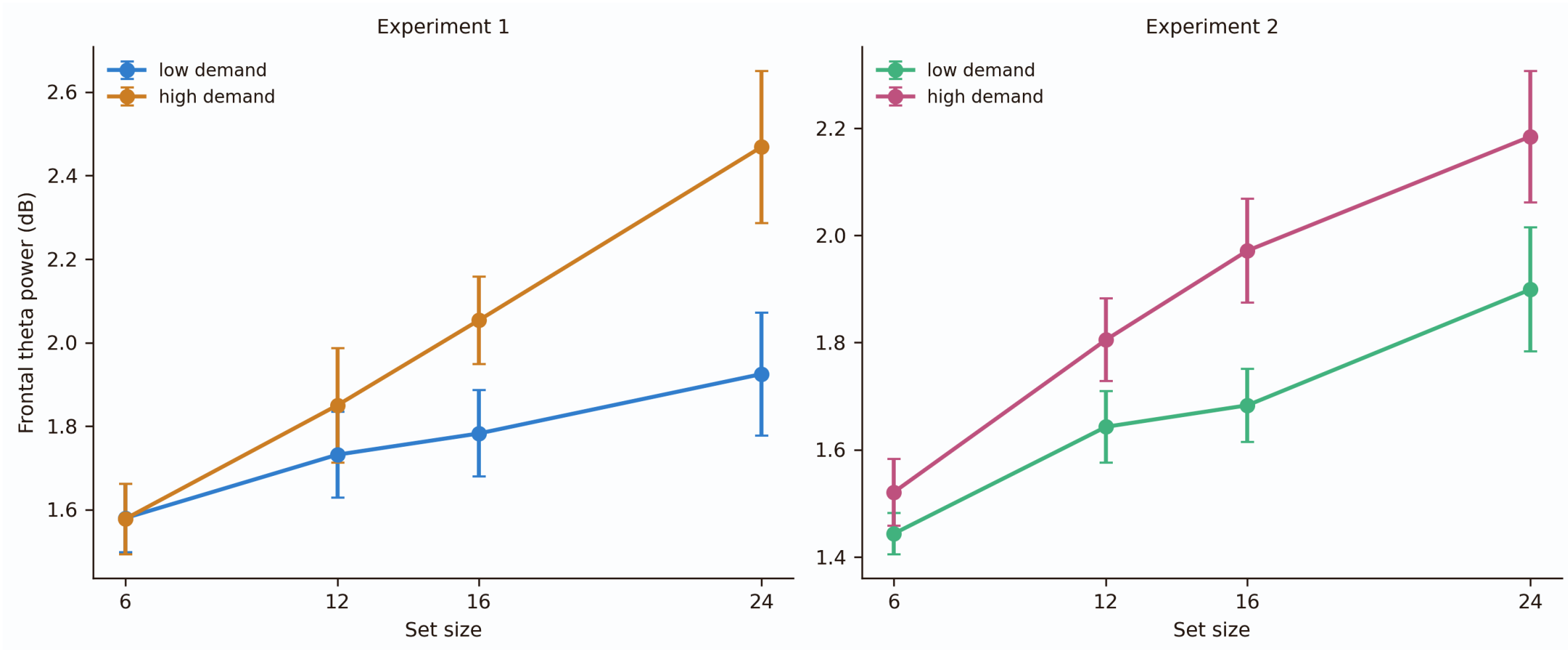


**Figure S3. Frontal-midline theta by demand and set size, related to Figures 2 and 3.**

Fixation-locked frontal-midline theta (4–8 Hz) power increased with both demand and set size in both experiments, providing a spectral marker of cognitive control that complements the occipital alpha analysis. Data are represented as mean ± SEM.

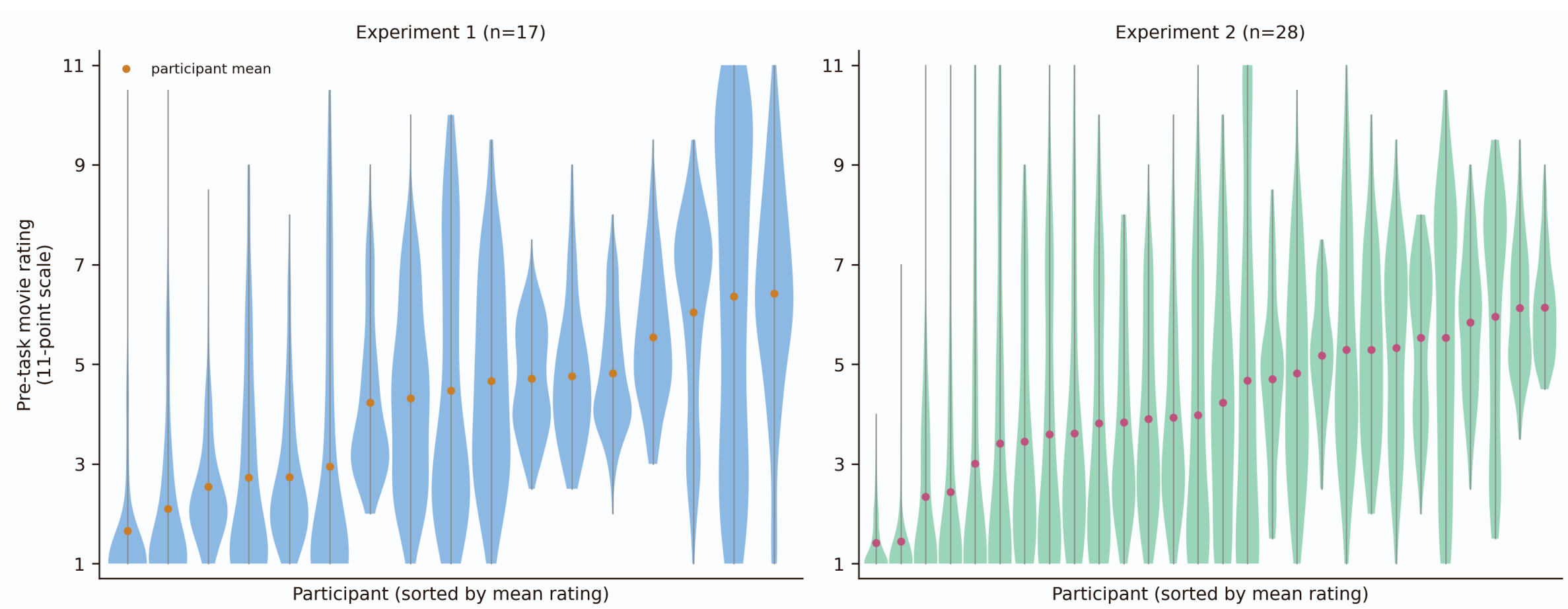


**Figure S4. Distribution of pre-task movie ratings, related to STAR Methods.**

Per-participant distributions of pre-task movie ratings for both experiments (each violin is one participant across ~189 unwatched posters; dot = participant mean, grey line = min–max range). Ratings span the 11-point scale and are well dispersed within every participant (mean within-participant SD = 1.83 [E1] and 2.04 [E2], spanning a mean within-participant range of 8.1 of the 11 scale points; the least-variable participant still had SD = 1.08 [E1] / 0.65 [E2]), confirming that participants discriminated among posters rather than rating uniformly.

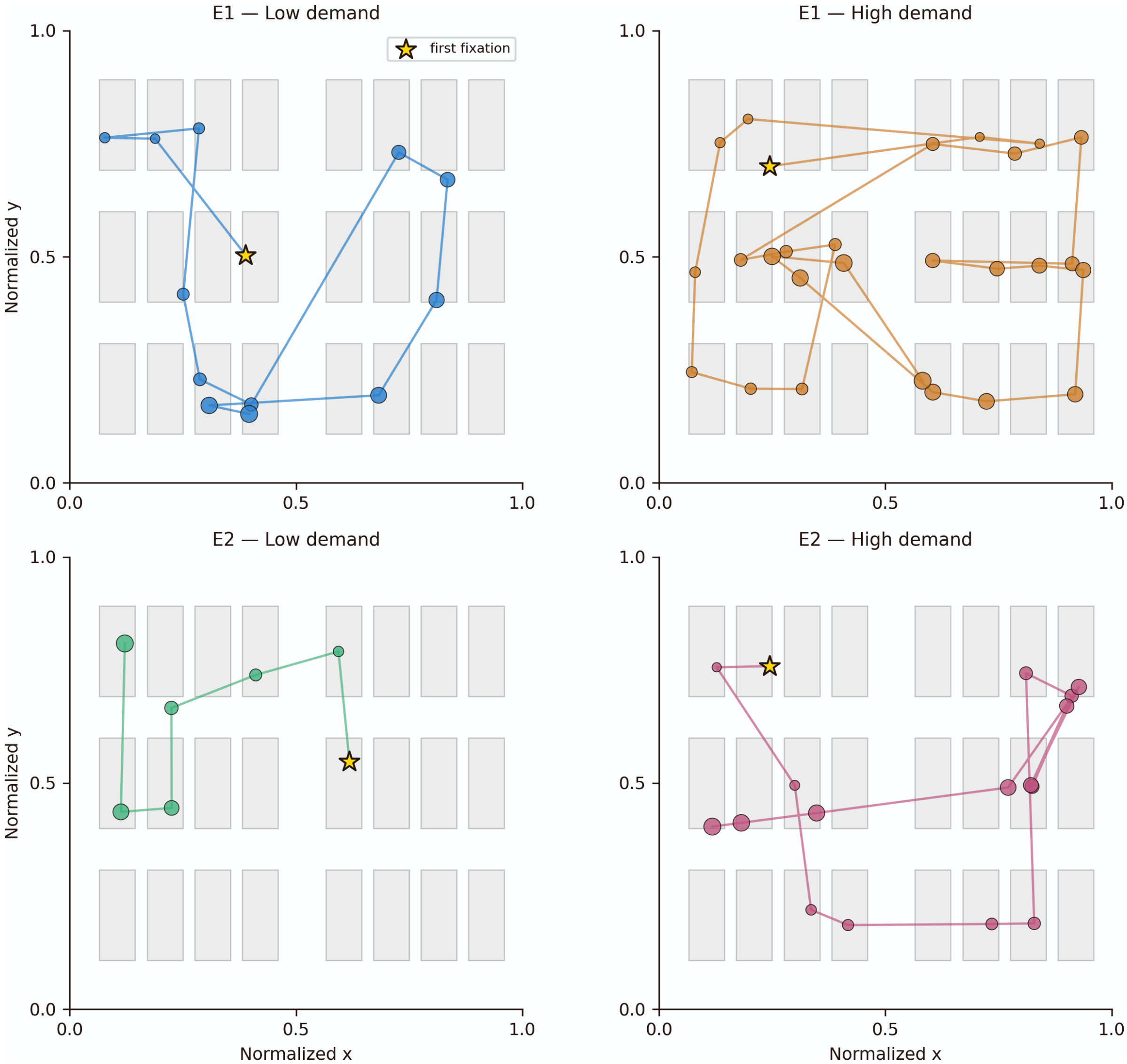


**Figure S5. Representative gaze scanpaths (trajectories) by demand condition, related to Figures 2 and 3.**

Example fixation sequences for low- and high-demand trials in each experiment; marker size grows with fixation order, illustrating the broader, more back-and-forth scanning under high demand. Grey boxes mark the fixed 3 × 8 poster slots of the choice array.
